# Influenza virus research in an era of emerging respiratory threats: host–virus interactions, surveillance technologies, antiviral management and resistance, and vaccine preparedness

**DOI:** 10.3389/fmicb.2026.1949447

**Published:** 2026-09-24

**Authors:** Mengnan Hu, Lei Liu, Guangli Yan, Le Yang, Guimin Zhang, Ling Kong, Yu Guan, Hui Sun, Yongxia Guan, Chang Liu, Ye Sun, Ying Han, Xijun Wang

**Affiliations:** 1 State Key Laboratory of Integration and Innovation of Classic Formula and Modern Chinese Medicine, National Chinmedomics Research Center, National TCM Key Laboratory of Serum Pharmacochemistry, Metabolomics Laboratory, Department of Pharmaceutical Analysis, Heilongjiang University of Chinese Medicine, Harbin, China; 2 State Key Laboratory of Dampness Syndrome, The Second Affiliated Hospital of Guangzhou University of Chinese Medicine, Guangzhou, China; 3 State Key Laboratory of Integration and Innovation of Classic Formula and Modern Chinese Medicine, Lunan Pharmaceutical Group Co. Ltd., Linyi, China

**Keywords:** antiviral resistance, emerging respiratory threats, host–virus interactions, influenza virus, surveillance technologies, vaccine preparedness

## Abstract

Seasonal epidemics, zoonotic spillover, antiviral resistance, and pandemic risk continue to make influenza a major challenge for respiratory preparedness. The recent spread of highly pathogenic avian influenza A(H5N1) in mammalian hosts has made it increasingly important to consider molecular adaptation, surveillance, treatment, and vaccination as connected parts of preparedness rather than as separate issues. This review focuses on four interconnected areas: host–virus interactions, surveillance technologies, antiviral management and resistance, and vaccine preparedness. Evidence on mammalian adaptation includes receptor specificity, viral polymerase–host factor compatibility, innate immune antagonism, ANP32 proteins, and aberrant viral RNA generation, while surveillance has expanded to multiplex and CRISPR-based assays, point-of-care platforms, genomic sequencing, artificial intelligence-assisted risk assessment, and environmental monitoring. These approaches differ in intended use and stage of validation. Antiviral benefit depends strongly on host risk and treatment timing. Resistance-associated substitutions, however, cannot be interpreted from sequence data alone and require phenotypic and epidemiological context. Vaccine development spans nanoparticle, mRNA, and intranasal platforms designed to broaden antigenic coverage, strengthen mucosal immunity, or improve protection in specific populations. Better analytical performance and stronger immune responses do not by themselves demonstrate reductions in severe disease, hospitalization, or mortality. Influenza preparedness ultimately depends on how effectively these four domains inform one another and how consistently emerging approaches are evaluated against clinically meaningful outcomes.

## Graphical abstract

**Figure:**
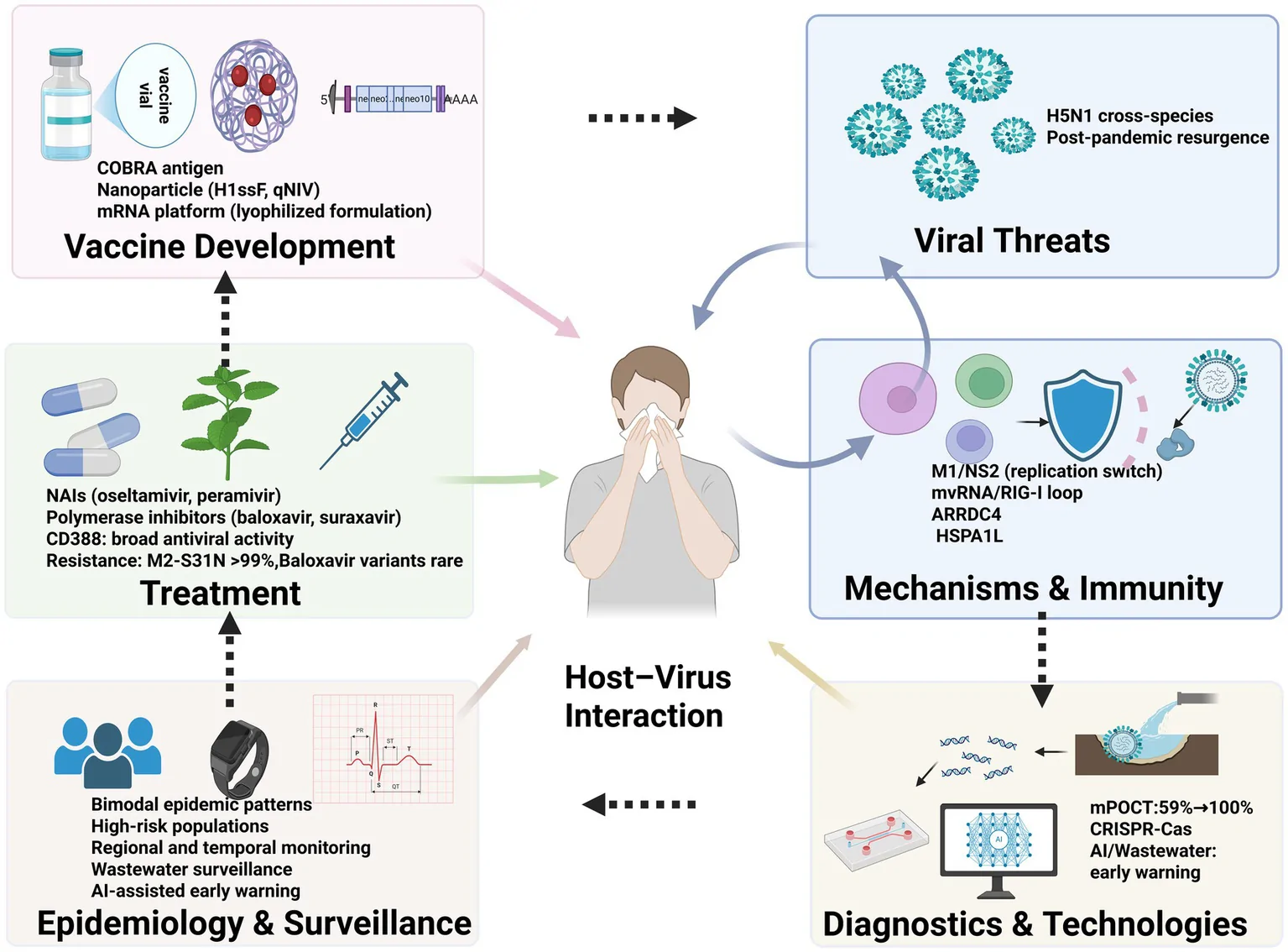
The central figure represents the host/patient. The surrounding modules summarize viral threats, pathogenic mechanisms, diagnostic and surveillance approaches, epidemiology and risk stratification, clinical management and recovery, and vaccine development. Examples shown include H5N1 threats; M1, NS2, mvRNA, and RIG-I-related mechanisms; multiplex PCR and CRISPR-based diagnostics; AI-assisted and wastewater surveillance; smartwatch-based recovery assessment; and COBRA, nanoparticle, and mRNA vaccine platforms. The converging arrows indicate the translational focus of the review: using information from these domains to improve protection of human health.

## 1 Introduction

The Global Burden of Disease (GBD) 2023 analysis estimated 2.50 million (95% uncertainty interval [UI], 2.24–2.81 million) lower respiratory infection (LRI) deaths worldwide in 2023 and approximately 22.5 million (19.6–26.5 million) LRI episodes attributable to influenza (GBD 2023 Lower Respiratory Infections and Antimicrobial Resistance Collaborators, 2026). Seasonal influenza remains a substantial component of the global respiratory-disease burden. At the same time, changes in influenza ecology, host range, and the circulation of other respiratory viruses have increased the importance of preparedness for zoonotic as well as seasonal threats.

Recent A(H5N1) activity provides a concrete example. Between March 2024 and May 2025, 70 human A(H5N1) infections were reported in the United States, mostly after exposure to infected dairy cattle or poultry; one fatal infection was linked to backyard poultry exposure (Rolfes et al., 2025). Infection of cats with highly pathogenic avian influenza A(H5N1) in South Korea provides additional evidence of the expanding mammalian host range (Kang et al., 2024). Continued circulation in mammals creates opportunities for further adaptation, so assessment of emerging influenza risk requires molecular information to be interpreted alongside One Health surveillance, clinical risk, antiviral preparedness, and vaccine development.

The difficulty is not a lack of recent advances in influenza virology, diagnostics, treatment, resistance surveillance, or vaccination, but the gap between performance in one domain and useful outcomes in another. Findings from cell or animal models may not predict human transmissibility or severity. Low analytical detection limits do not establish clinical utility, sequence-detected resistance markers do not by themselves establish clinically meaningful resistance, and stronger vaccine immunogenicity does not guarantee fewer severe outcomes. These limits are especially important when evidence is applied to older adults, immunocompromised patients, or populations exposed to emerging zoonotic viruses.

The review therefore follows four linked dimensions of influenza preparedness: (1) viral and host determinants of replication, mammalian adaptation, and immune responses; (2) multi-layer surveillance for early detection and risk assessment; (3) antiviral treatment, stewardship, and resistance monitoring; and (4) vaccine strategies for broad, durable, and population-specific protection. Epidemiological risk groups, clinical outcomes, and emerging technologies are included only when they inform at least one of these domains. They are not treated as additional pillars. This structure links mechanistic evidence with surveillance, therapeutic decisions, resistance monitoring, and vaccine preparedness in a single narrative.

### 1.1 Literature search strategy

PubMed/MEDLINE, Web of Science, and Scopus were searched for influenza studies published mainly from 2021 through 2026. Initial mapping used the broad concept “influenza,” followed by title-and-abstract screening. The narrative synthesis was limited to four preparedness-oriented domains: host–virus interactions, surveillance and diagnostic technologies, antiviral treatment and resistance, and vaccine preparedness. Earlier publications were retained when they provided essential mechanistic or clinical context. Priority was given to original research, clinical studies, large observational cohorts, surveillance reports, and publications from major public-health organizations.

## 2 Pathogenic mechanisms and host immunity

Influenza virus pathogenicity arises from complex host–virus interactions. Recent studies have revealed key mechanisms regulating viral replication, host immunity, and tissue repair (Figure 1).

**Figure 1:**
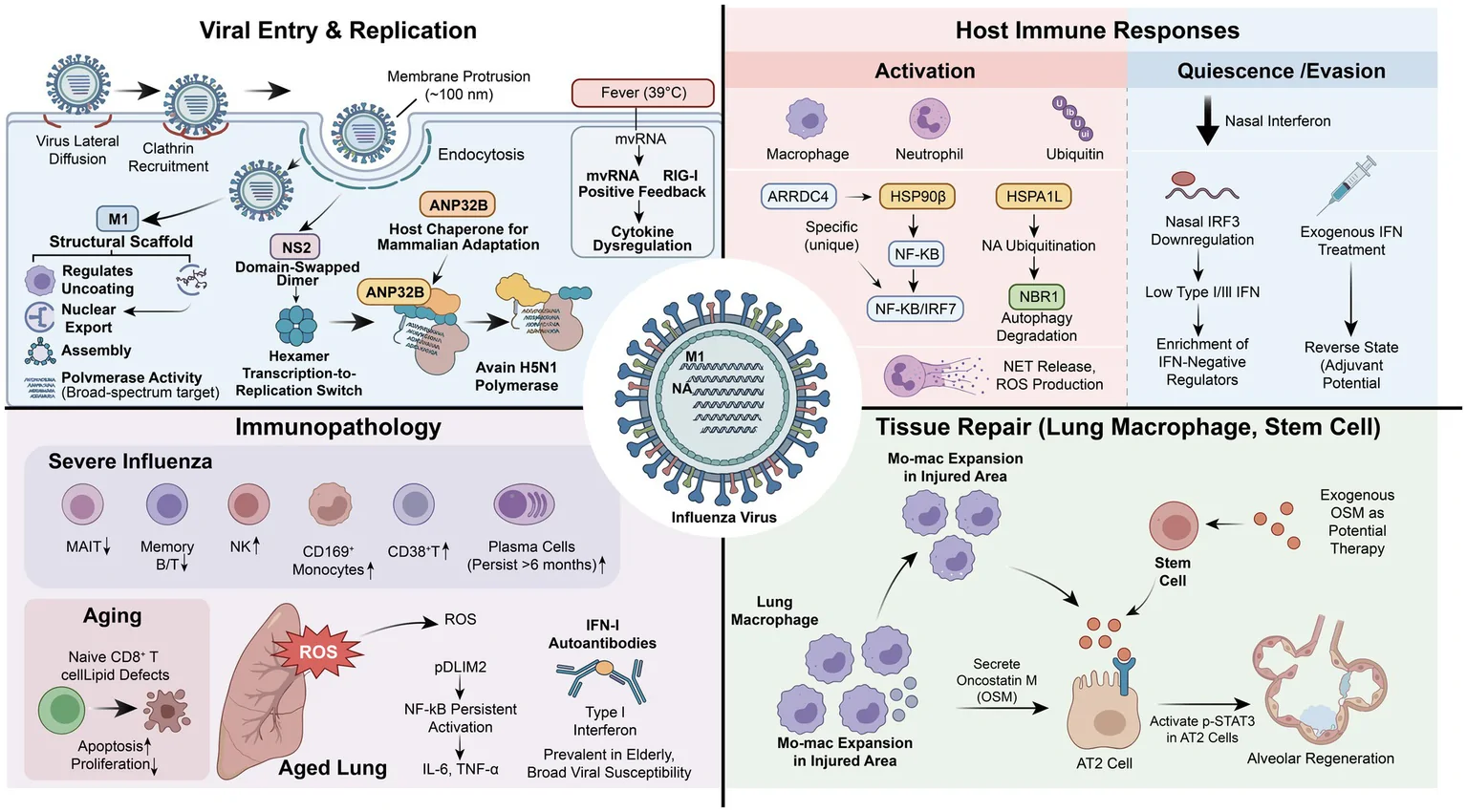
Schematic of influenza A virus pathogenesis, host immunity, immunopathology, and tissue repair. The diagram is divided into four quadrants around the virus. Top left: viral entry (lateral diffusion, clathrin recruitment, membrane protrusion, endocytosis) and replication regulation (M1, NS2, ANP32B, fever-induced mvRNA/RIG-I). Top right: host immune responses, including innate activation (ARRDC4/FBP/NF-κB, HSPA1L/NA ubiquitination, neutrophil NET/ROS) and nasal immune quiescence (IRF3↓, low IFN, reversible by exogenous IFN). Bottom left: immunopathology, including severe influenza-associated immune alterations (reduced MAIT-cell responses, increased NK-cell activity, and altered memory B/T-cell responses), aging-related CD8^+^ T-cell defects, the aged lung PDLIM2/NF-κB axis, and IFN-I autoantibodies. Bottom right: tissue repair via the Mo-Mac/OSM/p-STAT3 pathway promoting alveolar regeneration. Arrows indicate causal or temporal relationships.

### 2.1 Viral determinants of replication, mammalian adaptation, and entry

For avian influenza viruses, one of the first obstacles to productive infection in mammals is receptor use. Avian viruses preferentially recognize α2,3-linked sialic acids, whereas efficient infection of the human upper respiratory tract generally requires greater compatibility with α2,6-linked receptors (Thompson and Paulson, 2021). Receptor preference alone is not sufficient, however. Once entry is possible, the viral polymerase must also function efficiently in mammalian cells.

Efficient replication in mammalian cells also depends on polymerase compatibility. Adaptive changes in polymerase proteins, particularly PB2, can increase polymerase activity, replication efficiency, and viral fitness in mammalian hosts (Gilbertson et al., 2023). PB2-E627K and PB2-D701N are well-characterized mammalian-adaptive substitutions, with effects that vary by viral genetic background and host species (Peacock et al., 2023). Human ANP32B promotes the transition of avian-origin H5N1 polymerase from transcriptase to replicase activity by supporting formation of an asymmetric polymerase dimer, thereby facilitating genome replication in mammalian cells (Staller et al., 2024).

Non-structural protein 1 (NS1) suppresses antiviral signaling at several levels, including interferon induction and interferon-stimulated gene responses (Zhang et al., 2024). Variation in NS1 function can alter immune evasion, replication fitness, and host adaptation (Evseev and Magor, 2021).

PB1-F2, an accessory protein encoded by the PB1 segment, affects mitochondrial function and inflammatory signaling and has been linked to influenza virulence (Cheung et al., 2024). In some settings, it may also increase susceptibility to secondary bacterial infection (Mikušová et al., 2022). The magnitude and direction of these effects vary across viral strains and host backgrounds, so PB1-F2 is better treated as a context-dependent determinant than as a universal marker of pathogenicity.

Matrix protein 1 (M1) contributes to uncoating, viral ribonucleoprotein (vRNP) nuclear export, virion assembly, and regulation of polymerase activity. Its conservation and functional importance have made M1 an attractive broad-spectrum antiviral target, although M1-directed approaches remain largely preclinical (Zhu et al., 2025).

NS2 forms a domain-swapped dimer and organizes influenza A virus polymerase dimers into a barrel-shaped hexamer. This arrangement restricts interaction between viral polymerase and the C-terminal domain of host RNA polymerase II, favoring a replicase rather than a transcriptase state (Sun et al., 2024). The structure provides a mechanistic explanation for polymerase-state switching; antiviral application remains preclinical.

Febrile temperature can alter viral RNA synthesis. By disturbing the balance between viral nucleoprotein and polymerase expression, fever promotes production of aberrant viral RNAs, including mini viral RNAs (mvRNAs). These RNAs activate retinoic acid-inducible gene I (RIG-I) signaling and can amplify innate immune responses (Bisht et al., 2026). Whether fever-induced mvRNAs make a measurable contribution to disease severity in humans remains unresolved.

Real-time virus-viewing atomic force microscopy has visualized influenza A virus diffusion on the plasma membrane, clathrin recruitment, and membrane remodeling before endocytosis. Neuraminidase inhibition reduced lateral viral movement, whereas changes in cell-surface sialic acid density altered diffusion behavior (Yoshida et al., 2025). The relevance of these measurements for predicting antiviral efficacy still requires validation in physiologically relevant models.

Mammalian adaptation involves several barriers, including receptor use, polymerase compatibility, host-factor support, immune antagonism, and cell-entry dynamics. Receptor preference and PB2 adaptation remain important markers alongside ANP32-dependent polymerase function, NS2-mediated polymerase remodeling, and aberrant viral RNA generation. Most supporting evidence still comes from structural, cellular, or animal studies. These observations are insufficient for use as stand-alone predictors of sustained transmission or human disease severity.

### 2.2 Host restriction and innate immune regulation

Host restriction extends beyond canonical interferon pathways. Metabolic regulation, protein-quality control, neutrophil activity, and tissue-specific immune states can influence influenza replication. Most of these mechanisms have not been validated as clinical targets in human influenza.

During influenza infection, arrestin domain-containing protein 4 (ARRDC4) is upregulated and increases phosphofructokinase activity, raising fructose-1,6-bisphosphate (FBP) production. FBP stabilizes heat shock protein 90β and promotes NF-κB/IRF7 signaling; exogenous FBP also enhanced antiviral activity in experimental models (Li Y. et al., 2025). The pathway links glucose metabolism to innate antiviral signaling, but therapeutic efficacy and safety in patients remain unknown.

Heat shock protein A1L (HSPA1L) binds influenza neuraminidase (NA), promotes ubiquitination at lysine 242, and directs NBR1-dependent autophagic degradation. The NA-K242R substitution escapes this restriction and enhances viral replication (Yan et al., 2025). Its frequency in circulating viruses and effect on clinical outcomes remain unknown.

In a susceptible mouse model, delayed neutrophil recruitment, impaired neutrophil extracellular trap formation, and reduced reactive oxygen species production were associated with greater viral replication and broader pulmonary inflammation. Transfer of functionally competent neutrophils reduced viral burden and inflammatory spread (Gong et al., 2025). These results support a protective role for timely neutrophil activity in that model, while leaving open the possibility of immunopathology when neutrophil activation is excessive or poorly timed in human infection.

Nasal tissues can also enter an immune-quiescent state during influenza infection, with weak type I and type III interferon responses despite substantial viral burden. Reduced interferon regulatory factor 3 activity and enrichment of negative interferon regulators have been associated with this state. Experimental interferon supplementation reversed the phenotype, promoted viral clearance, and improved responses to intranasal vaccination by increasing dendritic-cell recruitment and tissue-resident memory T-cell generation (Sojati et al., 2025). Human applicability remains to be determined.

These studies broaden the concept of host restriction to include metabolism, proteostasis, innate-cell function, and local tissue state. The evidence is uneven, however: several of the most compelling mechanisms have been demonstrated in cells or animals rather than in patients. Translation will require human validation, safety assessment, and evidence that manipulating the pathway improves a clinically meaningful outcome.

### 2.3 Immunopathology, high-risk host factors, and tissue repair

Tissue injury during influenza reflects both viral replication and host responses. In older adults and other vulnerable populations, the timing and resolution of inflammation are especially important because immune dysfunction may persist after the acute infection.

High-dimensional single-cell analyses of severe seasonal influenza identified persistent changes in circulating immune-cell populations after clinical recovery. Reduced ICOS^+^ mucosal-associated invariant T cells, CXCR5^+^ memory B cells, and selected memory T-cell subsets were observed alongside increases in CD16^+^CD161^+^ natural killer cells, CD169^+^ monocytes, and CD38^+^ plasma cells; some differences persisted for up to six months (Bodin et al., 2025). Whether this prolonged remodeling alters reinfection risk, vaccine responsiveness, or post-infectious complications remains uncertain.

Age-related T-cell dysfunction has also been linked to metabolic changes. In older individuals, naïve CD8^+^ T cells showed increased apoptosis and reduced proliferation together with greater fatty-acid uptake, neutral-lipid accumulation, and elevated mitochondrial membrane potential. Rosiglitazone partially reversed these abnormalities experimentally (Nicoli et al., 2022). The result identifies a metabolic component of immune aging, but therapeutic manipulation to improve influenza immunity or vaccine response remains investigational.

The aging lung microenvironment may simultaneously amplify inflammatory injury. Reactive oxygen species accumulation in senescent cells can downregulate the E3 ubiquitin ligase PDLIM2, impair degradation of the NF-κB subunit p65, and sustain production of pro-inflammatory cytokines such as IL-6 and TNF-α. Restoration of PDLIM2 expression reduced excessive inflammation in aged mice (Tian et al., 2025). Together, these findings indicate that immunosenescence involves both impaired antiviral defense and exaggerated inflammatory signaling, which may help explain the disproportionate burden of severe influenza in older adults.

Pre-existing abnormalities in antiviral immunity may further identify populations at increased risk of severe disease. Neutralizing autoantibodies against type I interferons (IFN-I) are estimated to be present in more than 100 million individuals worldwide, but their prevalence is strongly age-dependent (Bastard et al., 2021; Groen and Hale, 2026). In a large study of uninfected individuals from the general population, autoantibodies neutralizing high concentrations of IFN-α and/or IFN-ω were detected in 0.18% of individuals aged 18–69 years, 1.1% of those aged 70–79 years, and 3.4% of those older than 80 years; when neutralization was assessed at lower, more physiological IFN concentrations, the prevalence among individuals older than 80 years reached 6.3% (Bastard et al., 2021). Thus, the global estimate of more than 100 million carriers represents an absolute population-level estimate, whereas the approximately 3–6% values refer to age-specific prevalence in individuals older than 80 years under different neutralization conditions. These autoantibodies can weaken IFN-I-mediated antiviral defense and have been associated with susceptibility to severe viral infections, including seasonal and zoonotic influenza (Groen and Hale, 2026). Their clinical relevance is not uniform: age, population, and neutralization threshold all matter, and routine screening cannot yet be recommended without evidence of predictive value, reproducibility, and clinical utility.

A murine H1N1 model combining lineage tracing, single-cell RNA sequencing, macrophage depletion, and alveolar organoid assays identified a macrophage–epithelial repair pathway after influenza lung injury. Monocyte-derived macrophages (Mo-Macs) accumulated in damaged alveoli and promoted proliferation of residual alveolar type II (AT2) epithelial cells. Mo-Macs secreted oncostatin M (OSM), activating JAK/STAT3 signaling and STAT3 phosphorylation in AT2 cells; macrophage depletion delayed repair, whereas exogenous OSM promoted AT2 sphere formation *in vitro* (Shang et al., 2025). Whether manipulating this Mo-Mac–OSM–STAT3 axis improves recovery in human influenza has not been tested.

The evidence in this section spans human observational studies and experimental animal models. Persistent immune remodeling, age-associated T-cell dysfunction, pulmonary inflammatory pathways, anti-IFN-I autoantibodies, and macrophage-mediated repair may all be relevant to risk stratification or recovery, but they differ substantially in their readiness for clinical application. Larger human studies are needed before these mechanisms can be used routinely for prediction or treatment.

## 3 Multi-layer surveillance for early detection and risk assessment

Influenza surveillance now serves several purposes beyond confirming individual cases. It must support subtype identification, resistance monitoring, outbreak detection, population-level trend assessment, and evaluation of zoonotic risk. No single platform covers all of these functions. Laboratory molecular assays, decentralized point-of-care tests, biosensors, AI-assisted interpretation, genomic analysis, and environmental monitoring therefore contribute at different points in the surveillance pathway, and their performance needs to be judged against the intended use and stage of validation.

### 3.1 Nucleic acid testing technologies

Multiplex reverse-transcription quantitative polymerase chain reaction (RT-qPCR) combines high analytical sensitivity with subtype-level differentiation and can detect influenza alongside other respiratory pathogens (Xu et al., 2026). Reverse-transcription droplet digital PCR (RT-ddPCR) provides absolute nucleic acid quantification without a standard curve and retains sensitivity at low viral concentrations, including in wastewater and other environmental samples (Zafeiriadou et al., 2025). Their surveillance applications overlap, but they address different analytical needs.

CRISPR-Cas platforms are being developed for rapid, portable detection. The amplification-free Craspase-NanoLock system couples CRISPR-associated protease activity to a NanoLock reporter (Xiao et al., 2025), while the Cas12a-based DETECTR assay uses isothermal amplification before CRISPR target recognition (Park B. J. et al., 2021). Both are rapid, but prospective validation across diverse populations and circulating variants is still needed before analytical performance can be equated with diagnostic accuracy.

RT-qPCR, RT-ddPCR, and CRISPR-based assays serve different surveillance roles. RT-qPCR supports routine confirmation and subtype characterization; RT-ddPCR is useful for low-concentration or environmental samples, and portable CRISPR assays may extend rapid testing beyond centralized laboratories. Intended use is a more appropriate basis for comparison than a single performance metric.

### 3.2 Isothermal amplification and microfluidic point-of-care devices

NASBA, RPA, and LAMP simplify molecular detection by avoiding repeated thermal cycling. They require relatively modest instrumentation and can achieve short reaction times, making them suitable for decentralized diagnostic settings.

Microfluidic systems can integrate specimen processing, reagent delivery, amplification, and signal detection. Recent examples include G4-NASBA fluorescence assays, fully automated rotary systems incorporating RT-LAMP, and pump-free RPA-based devices (Luo et al., 2026; Zhang D. et al., 2025; Wu et al., 2025). Some platforms support parallel reactions or sample-to-answer workflows; extraction-free RT-LAMP microfluidic devices can detect multiple respiratory viruses within approximately 30 min (Lim et al., 2024).

Shorter reaction times are useful when early antiviral treatment matters. Practical deployment also depends on contamination control, sample preparation, subtype coverage, manufacturing scalability, and reproducibility in routine clinical specimens.

### 3.3 Saliva-based detection and rapid Immunochromatographic assays

Saliva offers a non-invasive alternative to nasopharyngeal sampling. Extraction-free PCR and multiplex RT-LAMP have shown that influenza viral nucleic acids can be detected from saliva (Varughese et al., 2025; Kshirsagar et al., 2025). The same specimen can be used to characterize influenza-specific mucosal IgA and IgG responses, a research application distinct from acute diagnosis (Tsunetsugu-Yokota et al., 2022).

Decentralized testing can also use rapid immunochromatographic formats. One tri-channel system based on quantum-dot fluorescent microspheres was developed to detect influenza A, influenza B, and SARS-CoV-2 simultaneously (Huang et al., 2026). Such multiplexing may be useful when several clinically similar respiratory viruses circulate at the same time.

Viral nucleic acid detection, mucosal IgA/IgG measurement, and multiplex antigen screening answer different clinical or research questions. They should not be judged by a single benchmark. Prospective validation is still required before newer saliva-based or immunochromatographic assays can replace established molecular testing in high-risk patients or in cases where diagnostic accuracy could affect clinical management.

### 3.4 Biosensor technologies

Several biosensor formats achieve rapid detection by amplifying optical, electrochemical, or transistor-based signals. Surface-enhanced Raman spectroscopy (SERS) combined with deep-learning classification has distinguished multiple respiratory viruses and viral variants (Kang et al., 2025). Reusable electrochemical sensors using receptor-functionalized microbeads have achieved femtomolar analytical sensitivity (Park et al., 2025), whereas graphene field-effect transistor biosensors have generated signals within seconds at very low target concentrations (Kumar et al., 2023).

Very low analytical limits are attractive, but they do not guarantee performance in clinical specimens. That is the main translational test. Manufacturing reproducibility, matrix interference, standardized sample processing, cost, and prospective validation may matter more for preparedness than another incremental reduction in the analytical detection limit.

### 3.5 Artificial intelligence-assisted diagnosis and risk stratification

Artificial intelligence and machine-learning tools are being explored for several influenza tasks, including diagnosis, subtype differentiation, and prediction of severe disease. Deep-learning analysis of pharyngeal images has distinguished influenza-related clinical patterns, while models using routine blood parameters have differentiated influenza A from influenza B (Okiyama et al., 2022; Hu et al., 2025). A separate line of work uses clinical nomograms built from routinely available indicators to identify children at increased risk of critical influenza (Xiong et al., 2025).

These tools are most likely to support clinical decisions rather than replace confirmatory testing. They may assist triage, prioritize confirmatory testing, or flag patients at higher risk of deterioration when molecular results are delayed. Reported accuracy and AUROC depend on dataset composition, prevalence, case definitions, and validation strategy, and single-center retrospective models may not transfer across seasons, populations, or viral subtypes. Routine use still requires external and prospective validation, calibration, and evidence of clinical impact.

### 3.6 Environmental, digital, and population-level surveillance

Wastewater surveillance can capture influenza activity that is not fully represented by healthcare-based case reporting. Viral RNA concentrations in wastewater have tracked trends in clinically confirmed infections, supporting use as an additional community-level indicator (Lehto et al., 2024). The signal is independent of individual testing behavior but does not provide a direct count of human infections.

Several pre-analytical and analytical steps affect wastewater measurements, including collection, concentration, storage, nucleic acid extraction, normalization, and assay selection. Extracted nucleic acids can remain suitable for retrospective analysis for extended periods, whereas untreated wastewater is less stable during prolonged storage (Farkas et al., 2024). These differences make cross-site comparisons difficult unless sampling and reporting procedures are harmonized.

Source attribution becomes especially important when wastewater surveillance is applied to zoonotic influenza. Species-specific digital PCR can detect H5Nx clade 2.3.4.4b viruses in biological and environmental samples (Buttinger et al., 2025), but an H5Nx wastewater signal does not establish human infection or human-to-human transmission. Depending on the catchment, viral material may derive from people, infected animals, agricultural waste, or animal-processing activities. Environmental results therefore need to be interpreted with animal surveillance, occupational exposure data, clinical testing, and genomic sequencing.

Digital and administrative data can add another layer to surveillance. Generative AI-assisted screening of electronic health records has been used to flag possible avian-influenza exposure (Goodman et al., 2025), standardized ICD-10 definitions can support cross-country burden comparisons (Sanchez Ruiz et al., 2025), and Belgium’s Respi-Radar combines clinical, nursing-home, hospital, and wastewater indicators in a single monitoring system (De Muylder et al., 2025). These approaches are most useful when they complement, rather than duplicate, laboratory and epidemiological surveillance.

The central challenge for population surveillance is integration. Signals from wastewater, clinical testing, electronic records, genomic sequencing, and animal surveillance differ in scale, timing, and specificity. Their value depends on common definitions, transparent thresholds, and procedures for reconciling discordant signals before they are used for public-health decisions (Table 1).

**Table 1:** Comparison of technologies for influenza detection and surveillance.

Modality	Method	Intended use/setting	Evidence stage	Validation context	Performance metric (study-specific)	Deployment characteristics	Major advantages	Major limitations	Reference(s)
Nucleic acid testing	Multiplex RT-qPCR	Clinical diagnosis and differential detection	Clinical validation	Clinical comparison	Diagnostic sensitivity 99.81%; specificity 100%; overall concordance 99.84% for six viruses	1–2 h; laboratory-based high-throughput testing	High-throughput; sensitive; simultaneous pathogen detection	Requires laboratory equipment and trained personnel	Xu et al. (2026)
Nucleic acid testing	RT-ddPCR	Low-copy quantification; environmental/wastewater surveillance	Analytical validation	Analytical/environmental validation	Limit of detection (LOD): 1.4–2.9 copies/μL	~2 h; specialized laboratory workflow	Absolute quantification; useful at low concentrations	Higher cost and specialized equipment	Zafeiriadou et al. (2025)
Nucleic acid testing	CNC platform (Craspase + NanoLock)	Rapid decentralized molecular detection	Prototype/analytical validation	Early analytical validation	Analytical sensitivity: 250 fM; amplification-free detection	10 min; portable molecular testing	Rapid; portable; no amplification step	Requires broader clinical validation	Xiao et al. (2025)
Nucleic acid testing	DETECTR	Rapid molecular detection	Analytical validation	Analytical validation	LOD: 1 PFU/reaction	<1 h; isothermal molecular detection	Rapid; isothermal; high target specificity	Lower throughput; clinical validation required	Park B. J. et al. (2021)
Isothermal amplification / microfluidic POCT	G4-NASBA	Rapid molecular testing	Clinical / analytical validation	Clinical / analytical comparison	LOD: 3 copies/μL; 94.0% concordance with commercial kits	15 min; portable testing platform	Rapid; portable	Sample preparation remains necessary	(GBD 2023 Lower Respiratory Infections and Antimicrobial Resistance Collaborators, 2026)
Isothermal amplification / microfluidic POCT	FA-RMP	Automated point-of-care testing	Prototype validation	Prototype/technical validation	16 parallel reactions; up to four clinical samples	~30 min; sample-in–answer-out workflow	Portable; parallel testing capability	Development complexity and cost	Zhang D. et al. (2025)
Isothermal amplification / microfluidic POCT	Bioinspired microfluidic sensing platform	Low-cost decentralized testing	Clinical validation	Retrospective clinical validation	LOD: 0.18 copies/μL; sensitivity 93.2%; specificity 97.7%	~40 min; portable pump-free platform	Low-cost; portable	Requires broader prospective validation	Wu et al. (2025)
Isothermal amplification / microfluidic POCT	RT-LAMP microfluidic device	Multiplex point-of-care testing	Technical/early clinical validation	Technical/early clinical validation	Simultaneous detection of four respiratory viruses	~30 min; application in resource-limited settings	Extraction-free; suitable for decentralized testing	Requires further optimization and validation	Lim et al. (2024)
Saliva-based testing	Extraction-free SYBR Green PCR	Noninvasive molecular diagnosis	Clinical validation	Clinical evaluation	Sensitivity 90.3%	1–2 h; simplified saliva workflow	Noninvasive; simplified workflow	Lower sensitivity than some laboratory molecular assays	Varughese et al. (2025)
Saliva-based testing	Multiplex RT-LAMP	Decentralized saliva testing	Early validation	Early validation	Performance not directly reported	<30 min; non-invasive testing	Convenient; non-invasive	Broader clinical validation required	Kshirsagar et al. (2025)
Mucosal immune assessment	Saliva-based IgA/IgG assay	Vaccine/mucosal immunity evaluation	Immunological assessment	Immunological assessment	Measures influenza-specific salivary IgA/IgG responses	Several hours; immunological monitoring workflow	Noninvasive assessment of mucosal immunity	Not designed for acute diagnosis	Tsunetsugu-Yokota et al. (2022)
Rapid immunochromatography	Tri-channel immunochromatographic system	Multiplex decentralized screening	Early clinical validation	Early clinical validation	Reported sensitivity 100% in saliva in the study cohort	10–15 min; rapid multiplex screening	Noninvasive; rapid; multiplex detection	Requires independent and larger-scale validation	Huang et al. (2026)
Biosensor	SERS + deep learning	Multipathogen classification	Prototype validation	Prototype/analytical validation	>98% classification accuracy for 13 respiratory viruses/variants in the reported dataset	Seconds; instrument-based detection	Rapid multiplex discrimination	Specialized instrumentation; external validation needed	Kang et al. (2025)
Biosensor	Electrochemical sensor	Rapid analytical detection	Prototype validation	Prototype/analytical validation	Femtomolar detection limit; reusable for 50 measurements	Minutes; reusable sensor platform	Reusable; highly sensitive	Clinical performance and scalability require validation	Park et al. (2025)
Biosensor	Graphene FET biosensor	Ultra-rapid analytical detection	Prototype validation	Prototype/analytical validation	LOD: 50 ag/mL; response time ~10 s	~10 s; ultra-rapid analytical detection	Very rapid; very low analytical LOD	Cost, manufacturing, and clinical validation limitations	Kumar et al. (2023)
AI-assisted diagnosis	Deep learning using pharyngeal images	Clinical decision support	Clinical dataset validation	Clinical dataset validation	Model performance: AUROC 0.90	Real-time after image acquisition	Rapid decision support	Depends on imaging quality and external validation	Okiyama et al. (2022)
AI-assisted diagnosis	CatBoost using routine blood parameters	Influenza A/B differentiation	External validation	External dataset validation	Accuracy 0.913 for A + vs. A−/B − and 0.939 for B + vs. A−/B−	Real-time after data availability	Uses routine laboratory parameters	Depends on laboratory data and population generalizability	Hu et al. (2025)
AI-assisted risk stratification	Clinical nomogram	Prediction of critical pediatric influenza	Retrospective clinical study	Retrospective clinical study	Training-set AUC 0.905	Real-time after data availability	Uses routine clinical indicators	Requires prospective validation	Xiong et al. (2025)
Environmental surveillance	Wastewater qPCR	Community-level trend monitoring	Population surveillance	Population/environmental surveillance	Viral RNA levels correlated with clinically confirmed cases	1–2 days; community-level monitoring	Independent of individual testing behavior	Methodological standardization and source attribution issues	Lehto et al. (2024)
Environmental surveillance	Long-term wastewater storage	Biobanking and retrospective surveillance	Laboratory validation	Laboratory stability study	Extracted nucleic acids stable ≥2 years	Long-term storage application	Supports retrospective analysis	Raw wastewater less suitable for prolonged storage	Farkas et al. (2024)
Environmental surveillance	H5Nx-specific digital PCR	Zoonotic/environmental H5 surveillance	Analytical/environmental validation	Analytical/environmental validation	High sensitivity and specificity for clade 2.3.4.4b	Hours; biological and environmental monitoring	Applicable to biological and environmental samples	Detection does not establish source or human transmission	Buttinger et al. (2025)
Digital surveillance	Generative AI + human EHR review	Identification of potential zoonotic exposure	Operational surveillance	Operational/clinical surveillance	Rapid identification of potential exposure events	Real-time; automated case finding support	Supports automated surveillance	Dependent on completeness and quality of EHR data	Goodman et al. (2025)
Administrative surveillance	ICD-10-based SARI surveillance	Cross-system disease-burden monitoring	Multi-country surveillance	Multi-country surveillance	Identified COVID-19 and influenza cases using standardized definitions	Near-real-time surveillance	Supports harmonized surveillance across settings	Dependent on coding completeness and accuracy	Sanchez Ruiz et al. (2025)
Integrated population surveillance	Respi-Radar	Multi-source respiratory early warning	Population surveillance system	Population surveillance	Four-level risk framework	Near-real-time; integrated surveillance streams	Combines complementary surveillance sources	Requires sustained multi-source data sharing and harmonization	De Muylder et al. (2025)

LOD, limit of detection; PFU, plaque-forming unit; fM, femtomolar; ag/mL, attogram per milliliter; AUROC, area under the receiver operating characteristic curve; NASBA, nucleic acid sequence-based amplification; G4-ThT, G-quadruplex–thioflavin T; FA-RMP, fully automated rotary microfluidic platform; TCIS, tri-channel immunochromatographic system; SARI, severe acute respiratory infection; EHR, electronic health record. Performance metrics are study-specific and should not be interpreted as direct head-to-head comparisons. Analytical limits of detection, clinical sensitivity and specificity, AI discrimination metrics, and population-level surveillance correlations represent distinct endpoints and levels of validation.

No single platform covers the entire surveillance pathway. Prospective validation, cross-site standardization, genomic confirmation where needed, and stable performance across seasons and circulating variants remain necessary. For zoonotic influenza, clinical, animal, environmental, and genomic data are most informative when interpreted together within a One Health framework.

## 4 Epidemiological context and high-risk populations

Epidemiological context is included here because it determines how surveillance, treatment, vaccination, and healthcare resources are prioritized. Severe outcomes are concentrated in specific populations, while subtype circulation, prior exposure, and zoonotic events change over time. The following subsections therefore focus on burden, vulnerable groups, epidemiological dynamics, and complications only where those features alter preparedness decisions (Figure 2).

**Figure 2:**
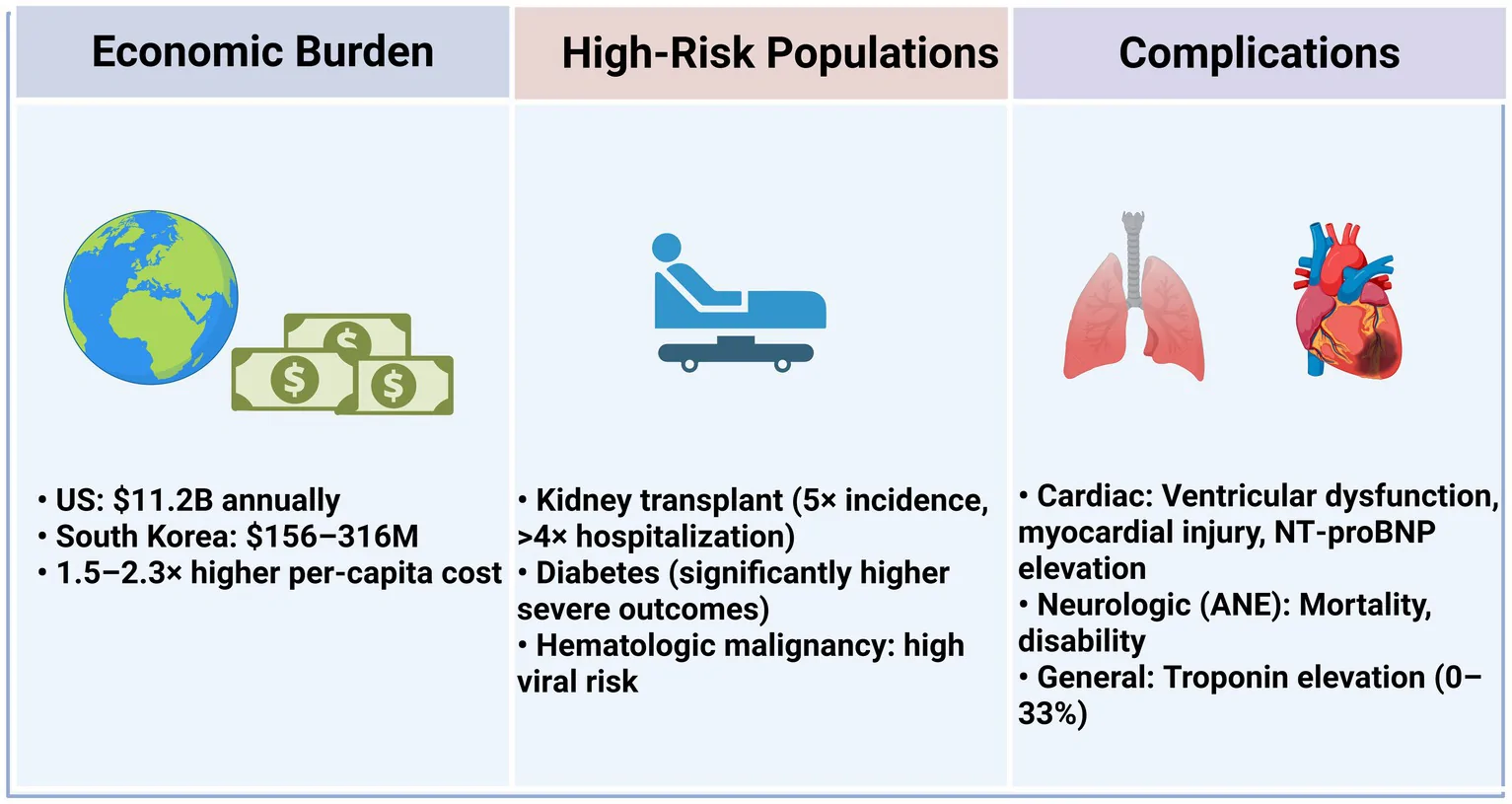
Conceptual illustration of influenza disease burden. The burden is divided into three categories: economic burden (annual costs in the US and South Korea, higher per-capita costs in elderly/comorbid individuals), high-risk populations (kidney transplant recipients, diabetics, hematologic malignancy patients), and complications (cardiac, neurologic, and troponin elevation). Created with BioRender.com.

### 4.1 Health and economic burden relevant to preparedness

In the (GBD 2023 Lower Respiratory Infections and Antimicrobial Resistance Collaborators, 2026) analysis, lower respiratory infections caused 2.50 million (95% UI, 2.24–2.81 million) deaths worldwide in 2023, and influenza was estimated to account for approximately 22.5 million (19.6–26.5 million) LRI episodes (2026). These figures support the use of influenza-specific information within broader respiratory-disease surveillance, particularly when identifying groups at higher risk of severe outcomes.

The economic burden of influenza extends beyond direct medical care. A United States analysis using 2015 data estimated an average annual economic burden of approximately US$11.2 billion, including US$8.0 billion in indirect costs (Putri et al., 2018). In South Korea, the annual socioeconomic cost of medically attended influenza among adults ranged from approximately US$156 million to US$316 million across five influenza seasons, while per-capita costs were 1.5–2.3 times higher among at-risk individuals than in the general adult population (Choi et al., 2025). Although such estimates vary by season, healthcare system, and costing methodology, they demonstrate that influenza burden includes substantial productivity loss and healthcare-resource use. Integrating burden estimates with virological surveillance and high-risk population profiles may therefore support targeted vaccination, antiviral allocation, and preparedness planning.

### 4.2 Burden in high-risk populations

Certain populations experience disproportionately severe influenza outcomes and should therefore be prioritized within prevention and treatment strategies. Kidney transplant recipients have been reported to experience approximately fivefold higher rates of laboratory-confirmed influenza and more than fourfold higher risks of hospitalization than the general population (Helanterä et al., 2021). Patients with hematologic malignancies are highly vulnerable to community-acquired respiratory viral infections. In a multicenter cohort including multiple respiratory viral pathogens, hospitalization occurred in 59.5% of patients and overall mortality reached 10.6% (Salmanton-García et al., 2025). Because those cohort estimates are not influenza-specific, they should be read as evidence of vulnerability to severe respiratory viral disease in profoundly immunocompromised hosts rather than as influenza-specific rates.

Influenza during pregnancy has been associated with increased risks of severe maternal morbidity, preterm birth, and hypertensive disorders, supporting timely vaccination, early diagnosis, and close clinical monitoring (Wen et al., 2021). Adults with diabetes have higher rates of influenza-related hospitalization, intensive care unit admission, pneumonia, mechanical ventilation, and in-hospital mortality than adults without diabetes (Owusu et al., 2022).

High-risk profiles have a direct practical use. They can guide vaccination, rapid diagnostic testing, and early antiviral treatment toward patients most likely to benefit, while also informing resource allocation during periods of intense influenza activity.

### 4.3 Epidemiological dynamics

Subtype dominance can change within a single season. Beijing in 2023–2024 showed a bimodal pattern with successive circulation of influenza A and influenza B viruses, and hospitalization was more frequent with influenza A than with influenza B (Zhu and Gu, 2024). Similar changes after COVID-19-related disruptions support tracking subtype prevalence together with clinical burden instead of relying on aggregate influenza activity.

After COVID-19-related interventions were relaxed, a prospective US pediatric study documented increases in binding and neutralizing antibodies to several respiratory viruses. The largest changes occurred in younger children who began with low antibody levels, and substantial exposure to SARS-CoV-2, enterovirus D68, respiratory syncytial virus, and influenza virus was observed (Nguyen-Tran et al., 2026).

Sequencing of A(H3N2), A(H1N1)pdm09, and B/Victoria viruses in South Tyrol identified distinct genetic clades and regional transmission patterns (Bianconi et al., 2025). Serological work provides a separate view of population immunity: early-life influenza B exposure can produce lineage-specific immune imprinting that may influence later susceptibility to B/Yamagata and B/Victoria viruses (Edler et al., 2024).

### 4.4 Influenza-associated complications and risk stratification

In children with influenza-associated acute necrotizing encephalopathy, mortality has been approximately 27%, and 63% of survivors had moderate-to-severe disability (Silverman et al., 2025). Neurological complications are uncommon but can be severe, and reliable prospective predictors remain limited.

Cardiac abnormalities have also been reported in patients hospitalized with influenza. In the FluHeart study, left- and right-ventricular dysfunction, elevated N-terminal pro-B-type natriuretic peptide (NT-proBNP), and myocardial injury were observed (Skaarup et al., 2025a). Across other influenza cohorts, troponin elevation has ranged from 0% to 33%, with higher frequencies generally reported in older adults, patients with comorbidities, and those with more severe disease (Lippi and Sanchis-Gomar, 2021).

Cardiovascular biomarkers or imaging may therefore be useful when cardiac involvement is clinically suspected, but the available evidence does not support routine cardiac screening for every patient with influenza. Studies are often retrospective, small, or regionally heterogeneous. Prospective work is needed to determine which markers add reproducible information to clinical risk stratification.

## 5 Antiviral treatment, stewardship, and resistance surveillance

This section focuses on the points at which antiviral choice, diagnostic timing, antimicrobial stewardship, and resistance surveillance intersect (Figure 3). Established therapies are separated from investigational approaches, and sequence-detected resistance markers are interpreted only in the context of phenotype, epidemiology, and antiviral exposure.

**Figure 3:**
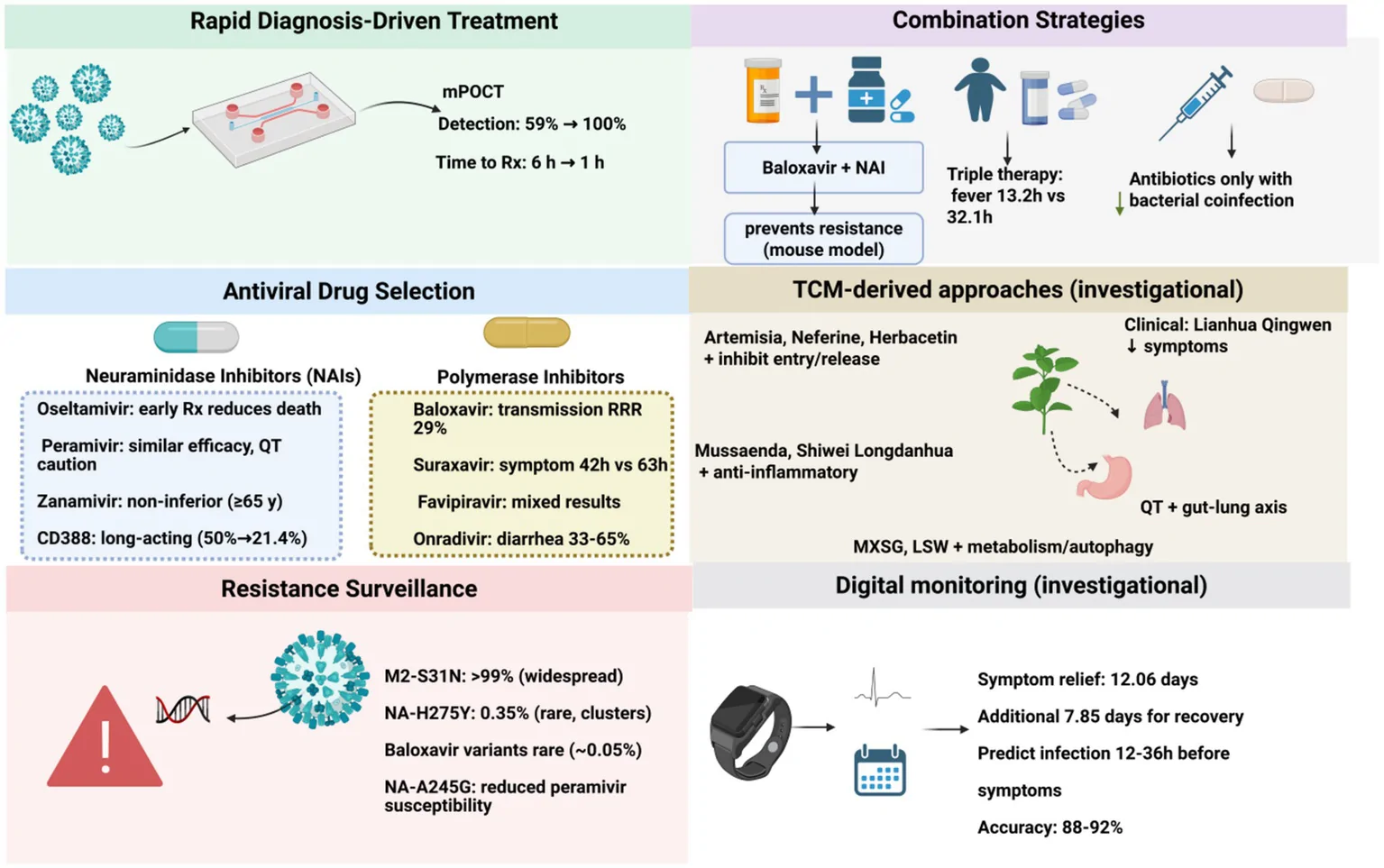
Integrated clinical management, antiviral stewardship, and resistance surveillance for influenza. Rapid molecular point-of-care testing can shorten the interval from diagnosis to antiviral treatment and support earlier infection-control measures. Neuraminidase inhibitors, including oseltamivir, zanamivir, and peramivir, remain established antiviral options, whereas polymerase-targeting agents and long-acting prophylactic candidates differ in indication, evidence maturity, and development status. Resistance-associated substitutions, including M2-S31N, NA-H275Y, NA-A245G, and baloxavir-associated PA substitutions such as PA-I38T, should be interpreted according to viral subtype, geographic and temporal context, antiviral exposure, phenotypic susceptibility, and clinical outcomes. Combination regimens, TCM-derived interventions, and wearable-based monitoring remain context-dependent or investigational and should not be considered routine standard-of-care approaches. Created with BioRender.com.

### 5.1 Neuraminidase inhibitors and long-acting prophylaxis

Surveillance data from 11 European countries showed that earlier neuraminidase inhibitor (NAI) treatment was associated with lower in-hospital mortality, with a greater apparent benefit among older patients (Adlhoch et al., 2023). Early treatment remains particularly important in hospitalized and high-risk patients, where rapid diagnosis and treatment access affect whether therapy can be started promptly.

Individual NAIs differ in route of administration, clinical setting, and safety considerations. In children with influenza A and bacterial coinfection, oseltamivir was associated with higher recovery rates and shorter hospital stays than peramivir (Xu et al., 2023). Inhaled zanamivir was non-inferior to oral oseltamivir for prevention of influenza-related hospitalization or death, including among older adults and patients with chronic lung disease (Su et al., 2022). A meta-analysis found no significant differences between intravenous peramivir and oral oseltamivir in complication rates, symptom alleviation, or fever reduction (Fang et al., 2021). Preclinical safety data have suggested QT-interval prolongation with peramivir in a canine model (Kambayashi et al., 2024); however, the clinical relevance of this finding in patients remains uncertain and should not be inferred directly from animal evidence.

Long-acting prophylactic agents may extend neuraminidase-targeted strategies beyond conventional acute treatment. CD388, a zanamivir–Fc conjugate designed for prolonged systemic exposure, demonstrated prophylactic activity in a controlled human influenza A(H3N2) challenge study. A single dose administered before viral challenge reduced the proportion of participants meeting the study definition of infection and decreased viral burden (Rojas et al., 2025). The challenge study supports the feasibility of extended-duration prophylaxis. Its role in routine influenza preparedness remains uncertain without evidence from real-world outbreaks, immunocompromised populations, and naturally acquired infection.

For neuraminidase inhibitors, timing and patient selection can matter as much as intrinsic antiviral activity. Route of administration and current susceptibility patterns also influence use. Rapid testing, risk stratification, timely prescribing, and resistance surveillance are most useful when they operate as a connected sequence.

### 5.2 Polymerase inhibitors and resistance-sensitive antiviral selection

Baloxavir marboxil inhibits the cap-dependent endonuclease, a mechanism distinct from neuraminidase inhibition. In uncomplicated outpatient influenza, it rapidly reduces viral load and shortens symptoms (Chong et al., 2021). Selected studies have also associated baloxavir use with less viral shedding and a lower risk of household transmission (Monto et al., 2025).

Treatment-emergent PA substitutions can occur after baloxavir use, particularly in pediatric outpatients. These substitutions were associated with delayed viral clearance, although clear worsening of clinical outcomes was not shown (Saito et al., 2025). Recurrent fever and PA-I38X substitutions were reported more often in children younger than 6 years (Hirotsu et al., 2023).

In patients hospitalized with severe influenza, adding baloxavir to standard-of-care neuraminidase-inhibitor therapy did not improve clinical outcomes compared with NAI treatment alone (Kumar et al., 2022). Routine baloxavir–NAI combination therapy is therefore not supported solely for clinical benefit in this population.

Several newer polymerase inhibitors are undergoing clinical evaluation, but their evidence maturity and safety profiles differ. In a phase 3 trial, the PA inhibitor suraxavir marboxil shortened the time to symptom alleviation compared with placebo, accelerated viral-load decline, and was associated with a low incidence of treatment-emergent PA-I38T substitutions (Wang et al., 2025). Favipiravir produced inconsistent clinical results across two phase 3 trials: one showed a reduction in symptom duration whereas the other did not meet its primary efficacy threshold, although antiviral activity was observed in both studies (Hayden et al., 2022). Onradivir, a PB2 inhibitor, demonstrated efficacy comparable to oseltamivir in a phase 3 trial but was associated with a substantially higher incidence of diarrhea than placebo (Yang et al., 2025).

The available evidence does not support a universal treatment hierarchy among these agents. Choice among polymerase inhibitors depends on disease severity, age, safety, treatment timing, resistance risk, and the susceptibility of circulating viruses. Table 2 summarizes the principal clinical and resistance features of current neuraminidase and polymerase inhibitors.

**Table 2:** Clinical evidence, safety, and resistance context of neuraminidase and polymerase inhibitors.

Drug class	Drug	Clinical evidence/setting	Main reported outcome	Safety/special population considerations	Resistance-associated findings	Interpretation	Reference(s)
Neuraminidase inhibitor	Oseltamivir	Hospitalized patients; pediatric influenza with bacterial coinfection	Earlier treatment associated with lower in-hospital mortality; improved recovery and shorter hospitalization reported in selected pediatric patients	Widely used in high-risk and hospitalized populations	NA-H275Y reported but uncommon in contemporary surveillance	Clinical benefit is greatest with timely treatment; resistance interpretation requires subtype and surveillance context	Adlhoch et al. (2023), Xu et al. (2023), Hussain et al. (2025), Patel et al. (2025)
Neuraminidase inhibitor	Peramivir	Comparative clinical studies/meta-analysis	Overall clinical efficacy broadly similar to oral oseltamivir for selected outcomes	QT prolongation observed in a canine model; human clinical significance uncertain	NA-A245G in influenza B associated with reduced susceptibility	Safety signals from animal studies should not be directly extrapolated to routine human use	Fang et al. (2021), Kambayashi et al. (2024), Patel et al. (2025)
Neuraminidase inhibitor	Zanamivir, inhaled	Comparative effectiveness	Non-inferior to oseltamivir for hospitalization/death outcome	Findings consistent in adults aged ≥65 years and patients with chronic lung disease	Not specifically reported in cited study	Useful alternative where inhaled administration is appropriate	Su et al. (2022)
Long-acting neuraminidase-targeted agent	CD388	Controlled human A(H3N2) challenge study	Single pre-exposure dose reduced infection and viral burden	Well tolerated in the challenge study	Not reported	Promising prophylactic strategy; real-world and high-risk population effectiveness remains unestablished	Rojas et al. (2025)
Polymerase inhibitor	Baloxavir marboxil	Uncomplicated outpatient influenza	Reduced viral load and symptom duration; reduced household transmission in selected studies	Treatment-emergent substitutions more frequent in young children	PA-I38X and other PA substitutions	Resistance selection is clinically relevant, particularly in pediatric populations	Chong et al. (2021), Monto et al. (2025), Saito et al. (2025), Hirotsu et al. (2023)
Polymerase inhibitor	Baloxavir + NAI	Severe hospitalized influenza	No additional clinical benefit over NAI treatment alone	Routine combination not supported for clinical benefit in severe hospitalized influenza	Resistance-prevention benefit not established clinically	Preclinical resistance findings should not be conflated with demonstrated clinical benefit	Kumar et al. (2022)
Polymerase inhibitor	Suraxavir marboxil	Phase 3 trial	Symptom alleviation 42.0 h vs. 63.0 h with placebo; faster viral decline	Generally acceptable study-specific safety profile	PA-I38T approximately 0.7–0.9% in reported study	Promising phase 3 evidence, but post-authorization surveillance remains important	Wang et al. (2025)
Polymerase inhibitor	Favipiravir	Two phase 3 trials	Clinical efficacy inconsistent; antiviral activity observed in both trials	Study-specific safety profile	Not reported	Antiviral activity did not translate consistently into the primary clinical endpoint	Hayden et al. (2022)
Polymerase inhibitor	Onradivir	Phase 3 trial	Clinical efficacy similar to oseltamivir	Diarrhea reported substantially more frequently than with placebo	Not reported	Efficacy must be balanced against tolerability	Yang et al. (2025)

NA, neuraminidase; PA, polymerase acidic protein; NAI, neuraminidase inhibitor. Resistance-associated substitutions should not automatically be interpreted as clinically meaningful resistance. Their significance depends on phenotypic susceptibility, viral fitness, transmission potential, treatment exposure, subtype, and epidemiological context. Outcomes shown in the table derive from different study designs and should not be interpreted as direct head-to-head comparisons.

Subtype, location, surveillance period, antiviral exposure, and phenotypic susceptibility provide the context needed to interpret sequence findings. M2-S31N remains common in contemporary seasonal influenza A, while NA-H275Y is uncommon in A(H1N1)pdm09. Baloxavir-associated PA substitutions, including PA-I38T and other sequence-flagged variants, likewise need treatment and phenotypic context (Takashita et al., 2025; Hussain et al., 2025; Patel et al., 2025).

### 5.3 Integrated clinical management and antiviral resistance surveillance

Compared with conventional testing, molecular point-of-care testing (mPOCT) increased laboratory confirmation of influenza and reduced the reported time to antiviral administration from about 6 h to 1 h, while also enabling earlier isolation and infection-control measures (Clark et al., 2021). The practical value is a shorter interval between presentation and an actionable decision.

In hospitalized children, clarithromycin–naproxen–oseltamivir was associated with shorter fever duration, faster viral-load decline, and no increase in reported adverse events compared with oseltamivir alone (Lee et al., 2021). In hospitalized influenza B, antiviral–antibiotic treatment was associated with fewer secondary bacterial infections, intensive-care admissions, and prolonged hospital stays than antiviral monotherapy (Ishaqui et al., 2021). Neither finding supports routine antibiotics for uncomplicated influenza. Antibacterial therapy should remain limited to suspected or confirmed bacterial coinfection.

In a mouse model, PA-I38X substitutions emerged in 67% of animals given baloxavir alone and in none receiving baloxavir–oseltamivir (Park J. H. et al., 2021). The result is relevant to resistance selection but remains hypothesis-generating for clinical practice because drug exposure, viral dynamics, and disease course differ between mice and patients. It does not override the absence of added clinical benefit from baloxavir–NAI combination therapy in severe hospitalized influenza (Kumar et al., 2022).

Continuous antiviral susceptibility surveillance is essential because resistance prevalence varies according to viral subtype, geographic region, antiviral exposure, and season. A sequence-based study of A(H1N1)pdm09 viruses collected from children in Shiraz, Iran, during 2015–2016 identified the H275Y substitution in 2 of 44 successfully sequenced isolates (Rashidi et al., 2021). This was a genotypic finding, and the small denominator limits inference regarding population-level prevalence or phenotypic resistance.

Contemporary surveillance further illustrates the need to distinguish genotypic markers from clinically meaningful resistance. During the 2023–2024 CDC/PAHO antiviral susceptibility surveillance program, sequence-based assessment included 5,123 viruses for M2 blockers, 6,874 for neuraminidase inhibitors, and 6,567 for baloxavir. More than 99% of influenza A viruses carried M2-S31N, whereas NA-H275Y remained uncommon (0.35%). Three viruses with reduced baloxavir susceptibility carried PA-I38T, PA-Y24C, or PA-V122A. Because the surveillance strategy relied primarily on genotypic screening, with supplementary phenotypic testing performed on approximately 7% of viruses, sequence-defined substitutions should not be interpreted as equivalent to clinical resistance without phenotypic and epidemiological context (Patel et al., 2025).

A resistance-associated substitution is only one part of the resistance phenotype. Its importance depends on the magnitude of susceptibility reduction, viral fitness, transmissibility, treatment history, subtype, and clinical outcome. Genomic surveillance is therefore most useful when paired, where feasible, with phenotypic susceptibility testing and epidemiological information.

Antiviral stewardship is best viewed as a feedback process: diagnosis informs drug selection, treatment outcomes and resistance-associated variants are monitored, and surveillance findings are used to refine later recommendations. The need for this linkage is greatest in hospitalized patients, young children, immunocompromised individuals, and other settings in which prolonged replication may favor resistance selection.

### 5.4 Traditional Chinese medicine as an adjunctive investigational approach

TCM-derived compounds and formulations have been studied as adjuncts to influenza treatment, with proposed effects on viral replication, inflammatory signaling, cellular metabolism, autophagy, and the gut–lung axis. The evidence is heterogeneous and predominantly preclinical. These approaches are therefore considered investigational adjuncts in this review, not alternatives to established antiviral therapy.

Experimental studies have reported direct antiviral effects for several plant-derived compounds. Artemisia-derived preparations have been linked to interference with hemagglutinin- and neuraminidase-related functions (Hu et al., 2026), neferine inhibited intracellular trafficking and nuclear translocation of influenza nucleoprotein (Ma et al., 2026), and herbacetin showed neuraminidase-targeting activity together with effects on post-infection pulmonary pathology (Liao et al., 2025). Pharmacokinetics, dosing, formulation consistency, and clinical effectiveness remain insufficiently characterized.

Other reported effects involve host-response pathways rather than direct viral targets. Experimental work has implicated TLR4/MyD88/NF-κB, PI3K–AKT, JAK/STAT, glycolysis-associated HMGB1 lactylation, and SPHK1/S1P signaling in individual compounds or formulations (Hu et al., 2026; Zhang J. et al., 2025; Yang et al., 2026; Feng et al., 2025; Ji et al., 2026; Lei et al., 2026). Gut–lung axis mechanisms have also been proposed, including changes in intestinal microbial composition and lipid metabolites with Qingjie Tuire Granules (Lu et al., 2026) and regulation of the IDO1–kynurenine pathway by chebulinic acid (Li R. et al., 2025). These observations come mainly from cellular or animal models and do not establish clinical efficacy.

Human evidence is much thinner. Randomized studies of Lianhua Qingwen Capsule have reported shorter recovery-related outcomes in some influenza populations (Shen and Yin, 2021), but differences in formulation, comparators, blinding, outcome definitions, and study design limit generalization. Bibliometric growth in this area (He et al., 2026) maps research activity; it does not establish therapeutic effectiveness.

Standardization and efficacy remain unresolved for TCM-derived interventions. Clinical studies need defined formulations and active components, pharmacological characterization, systematic safety monitoring, adequately powered blinded randomized designs, and clinically meaningful endpoints. Potentially toxic ingredients and interactions with conventional antivirals require separate assessment. TCM should remain an investigational adjunct until these requirements are met.

### 5.5 Digital monitoring as an investigational adjunct

Wearables have been studied for recovery monitoring and for detecting infection-related physiological changes before symptom onset. Smartwatch data suggest that physiological recovery can lag behind symptom resolution (Levi et al., 2026). Controlled viral-challenge studies have detected changes before symptom onset (Grzesiak et al., 2021). These studies involve small or selected populations and device-specific algorithms, and no prospective study has shown that wearable-guided management improves clinical outcomes. Digital monitoring remains exploratory and is not part of routine influenza care.

Antiviral management should be matched to patient risk and surveillance context. Early NAI therapy remains important for hospitalized and high-risk patients, while age, severity, safety, and resistance selection influence the use of polymerase inhibitors. Rapid molecular testing can shorten the path to treatment, and genomic and phenotypic surveillance can provide susceptibility feedback. TCM-derived approaches and wearable monitoring remain investigational.

## 6 Vaccine innovation and pandemic preparedness

“Next-generation” influenza vaccination addresses several distinct problems: antigenic breadth, mucosal immunity, weaker responses in selected populations, manufacturing flexibility, and pandemic readiness. The supporting evidence ranges from formulation studies and animal challenge models to randomized clinical trials. Sections 6.1–6.4 and Tables 3, 4 treat immunogenicity, protection, effectiveness, and storage stability as separate outcome domains.

**Table 3:** Developmental and formulation characteristics of selected influenza vaccine platforms and antibody-based strategies.

Candidate/strategy	Developer/source	Population/stage	Route/adjuvant	Dose/schedule	Formulation/storage	Reference
COBRA HA-based IIV (Y2/J4)	UGA/Cleveland Clinic-led academic study	DBA/2 J mice; preclinical immune/challenge	IM; WIV/SIV ± AddaVax	0.1 or 1 μg HA; weeks 0 + 4	Bivalent COBRA IIV; storage NR	Shi et al. (2024)
H1ssF ferritin nanoparticle	NIAID/NIH VRC	Healthy adults 18–70; phase 1	IM; ferritin nanoparticle; no adjuvant	20 μg once or 60 μg × 2; 16-week interval	Stabilized H1 HA-stem; storage NR	Widge et al. (2023)
Matrix-M-adjuvanted qNIV	Novavax	Adults ≥65; phase 3 randomized trial	IM; Matrix-M	60 μg HA/strain ×4 + 75 μg Matrix-M; single	Recombinant HA nanoparticle; storage NR	Shinde et al. (2022)
Mosaic H1N1 LAIV	CAMS/PUMC-led academic study	BALB/c mice; preclinical immune/challenge	Intranasal; live attenuated	10^7 EID50/30 μL × 2; 21-day interval	Mosaic HA/NA on Len/17 backbone; genetic stability evaluated	Ma et al. (2026)
H3N2 M2SR	FluGen	Adults 65–85; phase 1b randomized trial	Intranasal M2SR ± IM Fluzone HD	M2SR 10^9 TCID50; single	Replication-limited vaccine; frozen cryovials; Fluzone HD 2–8 °C	Eiden et al. (2024)
Lyophilized PR8 HA mRNA-LNP	UPenn/Genevant	Mice; preclinical formulation/immunogenicity	IM or ID; LNP	10 μg; single	Lyophilized; activity retained 12 wk. at RT and ≥24 wk. at 4 °C	Muramatsu et al. (2022)
High-dose/MF59 IIV (STOP-FLU)	Licensed VaxigripTetra/Fluad/Fluzone-HD	SOT adults; multicenter randomized trial	IM; MF59 in Fluad arm	15 μg/strain standard or MF59; 60 μg/strain high-dose; single	Commercial split-virion IIVs; storage NR	Mombelli et al. (2024)
MF59-adjuvanted TIV (aTIV)	NR in cited substudy	Nursing-home residents; immunogenicity substudy	aTIV vs. TIV; route NR	NR	Trivalent IIV; storage NR	Smith et al. (2023)
RIV4 (Flublok Quadrivalent)	Sanofi Pasteur	Healthcare personnel 18–64; randomized open-label trial	IM	45 μg HA/strain; 0.5 mL single dose	Recombinant HA; no NA antigen; storage NR	Dawood et al. (2021)
High-dose quadrivalent/trivalent IIV	Sanofi high-dose products + RCT meta-analysis	Adults ≥65; randomized effectiveness evidence	IM	60 vs. 15 μg HA/strain; single seasonal dose	Licensed high-dose IIV; storage NR	Johansen et al. (2025), Skaarup et al. (2025b), Leibovici Weissman et al. (2021)
LAIV-4	Licensed LAIV-4	Children 2–6; nationwide target-trial emulation	Intranasal	2 doses recommended if previously unvaccinated	Quadrivalent live attenuated vaccine; storage NR	Kildegaard et al. (2023)
MVA-NP + M1	Vaccitech/University of Oxford	Adults ≥18 after seasonal QIV; phase 2b randomized trial	IM; MVA vector	1.5 × 10^8 PFU; single	Replication-deficient vector expressing NP/M1; storage NR	Evans et al. (2022)
Broad H5Nx mAbs	NIH/NIAID VRC	Human-derived mAbs; lethal H5N1 mouse challenge	Passive transfer, i.p.	0.3–10 mg/kg; key leads 0.6–1 mg/kg	H5 HA-head mAbs; storage NR	Abu-Shmais et al. (2025)
HA/NA bispecific antibody	Technion-led academic study	*In vitro* + mouse models; preclinical	i.p.	2 mg/kg	HA/NA bispecific; mouse t1/2 ≈ 6.1 d; storage NR	Moirangthem et al. (2024)
MHAA4549A	Genentech	Adults with uncomplicated influenza A; phase 2 randomized trial	IV	3,600 or 8,400 mg; single, ≤72 h from onset	Anti-HA-stalk IgG1; storage NR	Lim et al. (2022)

**Table 4:** Immunological, protection, and translational outcomes of selected influenza vaccine platforms and antibody-based strategies.

Candidate/strategy	Evidence level/target	Humoral/mucosal response	Cellular response	Cross-protection/clinical outcome	Key limitation/interpretation	Reference
COBRA HA-based IIV	Level C; COBRA H1/H3 HA	Broad HAI/neutralization ~2 wk. post-boost; persistence to ~20 wk. post-boost	ASC/IgG-subclass data; no direct T-cell endpoint	Heterologous H1N1/H3N2 protection in mice	Animal-only; human efficacy unknown	Shi et al. (2024)
H1ssF nanoparticle	Level B; conserved group 1 HA stem	Cross-neutralizing H1/H2/H5 antibodies; detectable >1 y	NR	No clinical efficacy endpoint	Small phase 1; limited group-2 breadth	Widge et al. (2023)
qNIV	Level B; four seasonal HAs	Day-28 HAI; broader heterologous H3N2/B responses	Day-7 polyfunctional CD4 + responses (IFNγ, TNFα, IL-2, CD40L)	Clinical efficacy not tested	Immunogenicity should not be equated with clinical benefit	Shinde et al. (2022)
Mosaic H1N1 LAIV	Level C; mosaic H1/NA1	Day-35 HAI/MN plus nasal/BAL IgA	Day-35 CD4/CD8 cytokines; respiratory TRM/BRM	Complete tested H1N1 protection; partial H3N2 protection (20% survival)	Mouse-only; long-term durability unknown	Ma et al. (2026)
H3N2 M2SR	Level B; H3N2	Day-29 HAI/MN/NAI + nasal sIgA; HAI seroconversion 48% vs. 31% with HD alone	Day-15 IFNγ and granzyme-B responses	Clinical efficacy not assessed	Early phase; short immunogenicity follow-up	Eiden et al. (2024)
PR8 HA mRNA-LNP	Level C; PR8 HA	HAI at 4 wk.; immunogenicity retained after nonfrozen storage	NR	No challenge or clinical endpoint	Formulation study; no human efficacy/durability	Muramatsu et al. (2022)
High-dose/MF59 IIV	Level B; seasonal H1/H3/B	Day-28 HAI response: 42% standard, 60% MF59, 66% high-dose; follow-up to D180	NR	PCR-confirmed influenza similar across groups	SOT-specific; superior clinical efficacy not established	Mombelli et al. (2024)
MF59-adjuvanted TIV	Level B; seasonal antigens/NA	D28 HAI/MN; higher H3N2 anti-NA with aTIV; difference persisted at D180	Cellular responses broadly similar between aTIV and TIV	Clinical efficacy not tested in this substudy	Small, frail, repeatedly vaccinated cohort	Smith et al. (2023)
RIV4	Level B; recombinant seasonal HA	Higher 1-mo responses vs. egg-based IIV4; some GMT advantage at 6 mo	NR	Clinical effectiveness not a primary endpoint	Healthcare-personnel population; immunogenicity ≠ effectiveness	Dawood et al. (2021)
High-dose IIV	Level A; seasonal HA	Stronger immunogenicity than standard dose	NR	Meta-analysis: lower laboratory-confirmed influenza; hospitalization effects endpoint-dependent	DANFLU-2 primary endpoint nonsignificant; secondary findings require caution	Johansen et al. (2025), Skaarup et al. (2025b), Leibovici Weissman et al. (2021)
LAIV-4	Level B; seasonal LAIV	Not measured	Not measured	Hospital-contact VE 64.3%; admission VE 36.9% (CI crossed null)	Observational; H3N2-dominant season; few severe events	Kildegaard et al. (2023)
MVA-NP + M1	Level A; conserved NP/M1	No substantial HAI/MN boost	~3 × IFNγ ELISpot at D28; CD4/CD8 response; waned by week 26	No reduction in laboratory-confirmed influenza or ILI; trial stopped for futility	T-cell boost did not translate into clinical benefit	Evans et al. (2022)
Broad H5Nx mAbs	Level C/D; H5 HA head	Broad H5Nx neutralization; 6-mo-derived mAbs generally more potent than 2-wk isolates	N/A	Strong prophylactic protection in lethal H5N1 mouse challenge	No human efficacy; escape and transmission protection unresolved	Abu-Shmais et al. (2025)
HA/NA bispecific antibody	Level C/D; HA + NA	Enhanced dual neutralization and Fc-effector activity	Fc-effector activation; vaccine T-cell endpoint N/A	Improved mouse survival/viral burden, including therapeutic use	H1N1-focused preclinical evidence; escape remains possible	Moirangthem et al. (2024)
MHAA4549A	Level A; influenza A HA stalk	Passive mAb; vaccine-type immunogenicity N/A	N/A	No improvement in symptoms, viral load, or shedding	Mild outpatients; treatment allowed up to 72 h after onset	Lim et al. (2022)

NR, not reported in the cited study; N/A, not applicable; HA, hemagglutinin; NA, neuraminidase; COBRA, computationally optimized broadly reactive antigen; qNIV, quadrivalent nanoparticle influenza vaccine; M2SR, M2-deficient single-replication influenza vaccine; LNP, lipid nanoparticle; RIV4, recombinant quadrivalent influenza vaccine; LAIV-4, quadrivalent live attenuated influenza vaccine. Evidence levels: Level A, randomized clinical efficacy/effectiveness studies with clinically meaningful endpoints; Level B, human clinical immunogenicity or observational studies; Level C, preclinical in vivo studies, including animal immunogenicity/challenge; Level D, in vitro or mechanistic studies. Immunogenicity, animal protection, clinical effectiveness, and formulation stability are distinct outcome domains and should not be interpreted as directly comparable measures of superiority.

### 6.1 Broadening antigenic coverage through conserved antigen design

COBRA design combines sequence information from multiple circulating strains to generate consensus-like hemagglutinin (HA) and neuraminidase (NA) immunogens intended to address antigenic drift. In animal models, COBRA-based constructs generated broadly neutralizing antibodies across multiple H1N1 and H3N2 clades and protected against antigenically diverse viruses (Shi et al., 2024). Durability in humans and clinical effectiveness remain untested.

In a phase 1 trial, a ferritin nanoparticle displaying an H1 HA stem immunogen (H1ssF) induced cross-neutralizing antibodies against group 1 viruses, including H1, H2, and H5 (Widge et al., 2023). Stem-directed antibodies may be less sensitive to antigenic drift than responses focused on the immunodominant HA head. Whether that breadth produces durable protection against clinically important outcomes still requires efficacy trials.

COBRA and HA-stem approaches both seek broader antigenic coverage, but they are supported by evidence at different stages. Durability remains uncertain, and clinical protection against antigenically divergent circulating viruses has not been established for either strategy.

### 6.2 Platform and delivery innovation for rapid and targeted immunization

Nanoparticle systems can modify antigen presentation, intranasal platforms can target respiratory mucosal immunity, and nucleic acid approaches can shorten redesign and manufacturing timelines. In older adults, a Matrix-M-adjuvanted quadrivalent nanoparticle vaccine produced stronger humoral and cellular responses than a conventional quadrivalent vaccine (Shinde et al., 2022). This is an immunogenicity advantage, not evidence of fewer severe clinical outcomes. Experience with manufacturing flexibility during the COVID-19 pandemic has increased interest in analogous influenza nucleic acid platforms.

Intranasal vaccination targets the respiratory portal of entry. A mosaic H1N1 live attenuated influenza vaccine induced systemic and mucosal immune responses and protected mice against homologous and heterologous influenza A viruses (Ma et al., 2025). Human evidence is at an earlier stage: in adults aged 65–85 years, intranasal M2-deficient single-replication vaccine (M2SR) given with a high-dose inactivated vaccine enhanced antibody responses and was well tolerated in a phase 1b study (Eiden et al., 2024). Whether stronger local immunity reduces infection, transmission, or severe disease remains a clinical question.

Rapid sequence redesign is a major attraction of mRNA platforms when new strains emerge, but influenza candidates remain largely in early clinical development and have not established effectiveness against seasonal or pandemic disease. Formulation raises a separate problem. In preclinical testing, thin-film freeze-drying produced a dry-powder HA mRNA–lipid nanoparticle formulation that retained stability under non-frozen storage conditions (Muramatsu et al., 2022). Better storage could simplify deployment; it is not evidence of immunological or clinical efficacy.

Nanoparticle, mucosal, and mRNA platforms address different problems—antigen presentation, local respiratory immunity, and manufacturing adaptability. Each platform should be evaluated according to the problem it is intended to address, rather than through direct comparison of unrelated endpoints.

### 6.3 Vaccination in older and high-risk populations

Older adults, transplant recipients, and other immunocompromised populations may respond differently to vaccination because of immunosenescence, immunosuppressive therapy, comorbidity, and impaired cellular immunity. Strategies used in these groups include higher antigen doses, adjuvants, recombinant antigen production, and alternative delivery approaches.

In solid organ transplant recipients, high-dose and MF59-adjuvanted formulations generated stronger immune responses than standard-dose vaccination (Mombelli et al., 2024). MF59-adjuvanted vaccination also improved anti-neuraminidase responses in nursing-home residents (Smith et al., 2023). Recombinant production avoids egg-adaptive changes that can alter vaccine antigens; recombinant quadrivalent vaccine showed improved immunogenicity compared with egg-based vaccine in healthcare personnel (Dawood et al., 2021). These studies address different populations and endpoints and are not direct comparisons of the platforms.

The DANFLU-2 trial illustrates why immunogenicity and clinical protection must be separated. Hospitalization for influenza or pneumonia occurred in 0.68% of high-dose recipients and 0.73% of standard-dose recipients (relative vaccine effectiveness [rVE], 5.9%; 95.2% CI, −2.1 to 13.4; *p* = 0.14), so the primary endpoint was not significantly reduced. Influenza-specific hospitalization was lower with high-dose vaccination (0.06% vs. 0.11%; rVE, 43.6%; 95.2% CI, 27.5–56.3), but the negative primary endpoint meant that secondary endpoints were not formally hypothesis tested and should be interpreted descriptively (Johansen et al., 2025; Skaarup et al., 2025b). A separate meta-analysis found fewer laboratory-confirmed A(H3N2) infections with high-dose trivalent vaccine but no significant reduction in overall hospitalization or mortality (Leibovici Weissman et al., 2021).

Effectiveness also varies by subtype and host. Across age groups, vaccine effectiveness has generally been lower against A(H3N2) than against A(H1N1)pdm09 (Shinjoh et al., 2021). A European multicenter study reported particularly limited effectiveness in people with kidney disease or immunosuppression (Rose et al., 2025). In other populations, quadrivalent live attenuated vaccine showed effectiveness against influenza-related hospitalization in young children (Kildegaard et al., 2023), and recombinant vaccine showed acceptable effectiveness in adolescents (Folegatti et al., 2025).

Host characteristics can alter vaccine-response profiles without producing a uniform loss of protection. In the cited studies, obesity and smoking were not consistently associated with lower antibody responses or outpatient vaccine effectiveness (King et al., 2024; Choi et al., 2024). Children with metabolically unhealthy obesity had altered post-vaccination cytokine responses despite antibody titers comparable with those of metabolically healthier children (Kainth et al., 2022). The discordance between cytokine and antibody readouts is a reminder that serology captures only part of the vaccine response.

Protection is also time-dependent. Vaccine effectiveness declines within approximately one year and may wane faster against A(H3N2) and influenza B than against A(H1N1)pdm09 (Nazareth et al., 2025). Antibodies carried over from the previous season provide only partial protection (Praphasiri et al., 2023). These findings continue to support annual vaccination even when enhanced-dose, adjuvanted, recombinant, or other improved formulations are used.

In vulnerable populations, evaluation therefore needs both immune readouts and clinical endpoints. Hospitalization, severe disease, and mortality are also influenced by immunosenescence, frailty, comorbidity, and impaired T-cell responses, which can weaken the correspondence between antibody measures and clinical protection.

### 6.4 Pandemic vaccine strategies and antibody-based countermeasures

Universal vaccine approaches targeting conserved internal proteins aim to provide protection before a strain-matched pandemic vaccine can be widely deployed. In the phase 2b FLU009 trial, MVA-NP + M1 did not add protection beyond seasonal vaccination (Evans et al., 2022). The negative clinical result shows why conserved-antigen immune responses cannot be assumed to translate into clinical benefit.

Antibody-based countermeasures offer another route to early pandemic protection. Monoclonal antibodies isolated from people vaccinated against H5N1 about 15 years earlier retained broad neutralizing activity against contemporary H5Nx clade 2.3.4.4b viruses and showed greater protective activity than an existing HA-stem antibody in mice (Abu-Shmais et al., 2025). Bispecific antibodies directed at both HA and NA also improved antiviral activity over combinations of individual monoclonal antibodies and altered escape profiles *in vitro* and *in vivo* (Moirangthem et al., 2024).

Broad neutralization *in vitro* or protection in animals does not establish therapeutic efficacy in patients. An HA-stem-targeting monoclonal antibody failed to improve outcomes in uncomplicated influenza A in a phase 2 trial (Lim et al., 2022). Prophylactic and therapeutic antibody use therefore need separate evaluation, with attention to timing, viral burden, tissue penetration, Fc-mediated activity, patient risk, and the endpoint being measured.

Advancement should be guided by intended use and the strength of the evidence rather than by the breadth of immune recognition alone.

To facilitate interpretation of the heterogeneous evidence summarized in Tables 3, 4, studies discussed in this review were considered according to translational evidence levels: Level A, randomized clinical efficacy or effectiveness studies with clinically meaningful endpoints; Level B, human clinical immunogenicity or observational studies; Level C, preclinical *in vivo* studies, including animal immunogenicity and challenge studies; and Level D, *in vitro* or mechanistic studies. Importantly, progression across these levels does not necessarily imply equivalent clinical relevance.

Evidence does not favor a single vaccine platform across all intended uses. COBRA and HA-stem designs emphasize antigenic breadth; intranasal approaches target mucosal immunity; mRNA platforms emphasize adaptability; and enhanced-dose, adjuvanted, or recombinant vaccines address performance in selected populations. These studies report different outcomes, including formulation stability, animal protection, and randomized clinical endpoints. Immunogenicity and durability should not be used as substitutes for infection, transmission, hospitalization, severe disease, or mortality.

## 7 Discussion

The evidence reviewed here points to a series of translational handoffs across the four domains. Molecular findings need confirmation in human-derived systems, and surveillance signals need enough standardization to support action. Antiviral susceptibility data require phenotypic and treatment context. Vaccine-induced immune responses must ultimately be assessed against clinically meaningful outcomes. Weakness at any transition can reduce the preparedness value of an otherwise promising approach.

Mammalian adaptation shows the distance between mechanistic insight and practical risk assessment. Receptor usage, viral polymerase regulation, ANP32-family proteins, NS1/NS2 function, and aberrant viral RNA generation have refined current models of cross-species adaptation. Much of the evidence still derives from structural studies, cultured cells, or animal models. Human infection adds variables such as age, pre-existing immunity, immunosenescence, fever, and other physiological conditions that can alter viral replication and host responses. Molecular adaptation markers remain candidate risk indicators until supported by human-derived experimental systems, clinical cohorts, and epidemiological evidence.

Surveillance signals vary in scale, source, and validation endpoint. Multiplex assays, CRISPR-based platforms, genomic sequencing, AI-assisted analysis, and wastewater monitoring cannot be judged by a single criterion. Sampling procedures, analytical thresholds, and reporting practices add further variation. Wastewater detection of A(H5N1) is especially difficult to interpret because viral material may reflect human shedding, infected animals, or animal waste. Environmental findings are most informative when combined with clinical testing, genomic characterization, epidemiological investigation, and veterinary surveillance within a One Health framework.

Clinical management has a similar interpretive constraint. A resistance-associated sequence change is not synonymous with clinical resistance. Phenotypic susceptibility, antiviral exposure, viral subtype, fitness, and treatment outcome remain necessary for judging its significance. Host-directed and TCM-derived interventions are harder to compare because study design, intervention standardization, and quality control vary. Wearable monitoring may capture longitudinal recovery patterns, but useful clinical thresholds and incremental benefit over routine assessment have not been established. These adjunctive and monitoring approaches remain investigational.

Vaccine studies make the gap between immune readouts and clinical benefit especially clear. COBRA antigens, nanoparticle vaccines, mRNA platforms, and intranasal approaches address antigenic mismatch, manufacturing speed, or weak mucosal immunity. Broader antibody responses or higher neutralizing titers do not necessarily mean fewer hospitalizations or less severe disease. In older or immunocompromised populations, immunosenescence and impaired cellular immunity can weaken the relationship between serological responses and protection. Evaluation should include humoral, cellular, and mucosal responses, durability, and clinically meaningful outcomes in the intended population.

Influenza preparedness depends on how these links function in practice. Evidence of viral adaptation can inform surveillance and risk assessment, and surveillance can guide clinical and public-health responses. Antiviral use generates information relevant to resistance monitoring. Viral evolution and changing population immunity influence vaccine development and updating. Standardized validation, transparent evidence grading, interoperable data sharing, and evaluation in high-risk populations are needed throughout the pathway. During seasonal epidemics, zoonotic outbreaks, or pandemics, the reliability of these links may matter more than the performance of any single technology.

## Glossary

ANP32B: acidic nuclear phosphoprotein 32 family member B
ARRDC4: arrestin domain-containing protein 4
AT2: type II alveolar epithelial cells
CA: chebulinic acid
DHA: docosahexaenoic acid
FBP: fructose-1,6-bisphosphate
G4-ThT: G-quadruplex–thioflavin T
HAdV: human adenovirus
HMPV: human metapneumovirus
HRV: human rhinovirus
HSP90β: heat shock protein 90 beta
HSPA1L: heat shock protein A1L (heat shock 70 kDa protein 1 L)
HTH: high temperature and humidity
LOD: limit of detection
LQC: Lianhua Qingwen Capsule
LSW: Liu Shen Wan
MAIT: mucosal-associated invariant T cells
mPOCT: molecular point-of-care testing
Mo-Mac: monocyte-derived macrophage
MXSG: Ma Xing Shi Gan Decoction
MyD88: myeloid differentiation primary response 88
NBR1: neighbor of BRCA1 gene 1
NET: neutrophil extracellular trap
NP: nucleoprotein
OSM: oncostatin M
PDLIM2: PDZ and LIM domain protein 2
QT: Qingjie Tuire Granules
RIG-I: retinoic acid-inducible gene I
RPA: recombinase polymerase amplification
RRR: relative risk reduction
RT-LAMP: reverse transcription loop-mediated isothermal amplification
SARI: severe acute respiratory infection
SPHK1: sphingosine kinase 1
S1P: sphingosine-1-phosphate
TCIS: tri-channel immunochromatographic system
1D-CNN: one-dimensional convolutional neural network
VE: vaccine effectiveness

## References

[ref1] Abu-Shmais A. A. Freeman G. Creanga A. Vukovich M. J. Malla T. Mantus G. E. . (2025). Cross-neutralizing and potent human monoclonal antibodies against historical and emerging H5Nx influenza viruses. Nat. Microbiol. 10, 2903–2918. doi: 10.1038/s41564-025-02137-x, 41087744 PMC12578633

[ref2] Adlhoch C. Delgado-Sanz C. Carnahan A. Larrauri A. Popovici O. Bossuyt N. . (2023). Effect of neuraminidase inhibitor (oseltamivir) treatment on outcome of hospitalised influenza patients, surveillance data from 11 EU countries, 2010 to 2020. Euro Surveill. 28:2200340. doi: 10.2807/1560-7917.ES.2023.28.4.2200340, 36700868 PMC9881178

[ref3] Bastard P. Gervais A. Le Voyer T. Rosain J. Philippot Q. Manry J. . (2021). Autoantibodies neutralizing type I Ifns are present in ~4% of uninfected individuals over 70 years old and account for ~20% of COVID-19 deaths. Sci. Immunol. 6:eabl4340. doi: 10.1126/sciimmunol.abl4340, 34413139 PMC8521484

[ref4] Bianconi I. Manica M. Moroder E. Guzzetta G. Merler S. Poletti P. . (2025). Tracking seasonal influenza trends in South Tyrol during 2022/2023 using genomic surveillance data. Influenza Other Respir. Viruses 19:e70083. doi: 10.1111/irv.70083, 40143444 PMC11946919

[ref5] Bisht K. Weilandt D. R. Lamb C. H. Elshina E. Myhrvold C. Te Velthuis A. J. W. (2026). Febrile temperature activates the innate immune response by promoting aberrant influenza a virus Rna synthesis. Sci. Adv. 12:eaeb2700. doi: 10.1126/sciadv.aeb2700, 41481744 PMC12758553

[ref6] Bodin J. Tunheim G. Kristoffersen A. B. Herstad T. K. Vianello E. Haks M. C. . (2025). High-dimensional single-cell phenotyping unveils persistent differences in immune cell profiles between severe and moderate seasonal influenza. Front. Immunol. 16:1576861. doi: 10.3389/fimmu.2025.1576861, 40766325 PMC12322504

[ref7] Buttinger G. Petrillo M. Valastro V. Marciano S. Crimaudo M. D'amico V. . (2025). Novel (d)PCR assays for influenza a(H5Nx) viruses clade 2.3.4.4b surveillance. Euro Surveill. 30:2500183. doi: 10.2807/1560-7917.ES.2025.30.33.2500183, 40843521 PMC12372895

[ref8] Cheung P.-H. H. Yuen T.-L. Tang T.-T. Leung H.-Y. Lee T. T.-W. Chan P. . (2024). Age-dependent pathogenesis of influenza a virus H7N9 mediated through Pb1-F2-induced mitochondrial Dna release and activation of cgas-sting-Nf-κB signaling. J. Med. Virol. 96:e70062. doi: 10.1002/jmv.70062, 39569434

[ref9] Choi M. J. Choi W. S. Song J. Y. Cheong H. J. Noh J. Y. Wie S. H. . (2025). Estimating the clinical and economic burden of medically attended influenza in South Korea, stratified by age and comorbidity: a five-season hospital-based surveillance data, 2014/15-2018/19. PLoS One 20:e0317643. doi: 10.1371/journal.pone.0317643, 40961172 PMC12443274

[ref10] Choi W. S. Nowalk M. P. Moehling Geffel K. Susick M. Saul S. Lin C. J. . (2024). Smoking and serological response to influenza vaccine. Hum. Vaccin. Immunother. 20:2404752. doi: 10.1080/21645515.2024.2404752, 39400298 PMC11485682

[ref11] Chong Y. Kawai N. Tani N. Bando T. Takasaki Y. Shindo S. . (2021). Virological and clinical outcomes in outpatients treated with baloxavir or oseltamivir: a Japanese multicenter study in the 2019-2020 influenza season. Antivir. Res. 192:105092. doi: 10.1016/j.antiviral.2021.105092, 34052230

[ref12] Clark T. W. Beard K. R. Brendish N. J. Malachira A. K. Mills S. Chan C. . (2021). Clinical impact of a routine, molecular, point-of-care, test-and-treat strategy for influenza in adults admitted to hospital (Flupoc): a multicentre, open-label, randomised controlled trial. Lancet Respir. Med. 9, 419–429. doi: 10.1016/S2213-2600(20)30469-0, 33285143 PMC9764870

[ref13] Dawood F. S. Naleway A. L. Flannery B. Levine M. Z. Murthy K. Sambhara S. . (2021). Comparison of the immunogenicity of cell culture-based and recombinant Quadrivalent influenza vaccines to conventional egg-based Quadrivalent influenza vaccines among healthcare personnel aged 18-64 years: a randomized open-label trial. Clin. Infect. Dis. 73, 1973–1981. doi: 10.1093/cid/ciab566, 34245243 PMC8499731

[ref14] De Muylder G. Couvreur S. Stefani G. Brugerolles C. Dockx Y. Callies M. . (2025). Respi-radar: a tool to monitor respiratory infections, Belgium, winter season 2023/24. Euro Surveill. 30:2400756. doi: 10.2807/1560-7917.ES.2025.30.35.2400756, 40910226 PMC12413606

[ref15] Edler P. Schwab L. S. U. Aban M. Wille M. Spirason N. Deng Y. M. . (2024). Immune imprinting in early life shapes cross-reactivity to influenza B virus haemagglutinin. Nat. Microbiol. 9, 2073–2083. doi: 10.1038/s41564-024-01732-8, 38890491

[ref16] Eiden J. Fierro C. White A. Davis M. Rhee M. Turner M. . (2024). Safety and immunogenicity of the intranasal H3N2 M2-deficient single-replication influenza vaccine alone or coadministered with an inactivated influenza vaccine (Fluzone high-dose Quadrivalent) in adults aged 65-85 years in the Usa: a multicentre, randomised, double-blind, double-dummy, phase 1b trial. Lancet Infect. Dis. 24, 1118–1129. doi: 10.1016/S1473-3099(24)00351-7, 39004096

[ref17] Evans T. G. Bussey L. Eagling-Vose E. Rutkowski K. Ellis C. Argent C. . (2022). Efficacy and safety of a universal influenza a vaccine (Mva-np+M1) in adults when given after seasonal quadrivalent influenza vaccine immunisation (Flu009): a phase 2b, randomised, double-blind trial. Lancet Infect. Dis. 22, 857–866. doi: 10.1016/S1473-3099(21)00702-7, 35305317

[ref18] Evseev D. Magor K. E. (2021). Molecular evolution of the influenza a virus non-structural protein 1 in interspecies transmission and adaptation. Front. Microbiol. 12:693204. doi: 10.3389/fmicb.2021.693204 PMC8521145 34671321

[ref19] Fang Y. H. Hsu T. H. Lin T. Y. Liu C. H. Chou S. C. Wu J. Y. . (2021). Comparing intravenous peramivir with oral oseltamivir for patients with influenza: a meta-analysis of randomized controlled trials. Expert Rev. Anti-Infect. Ther. 19, 1039–1046. doi: 10.1080/14787210.2021.1878025, 33641583

[ref20] Farkas K. Fletcher J. Oxley J. Ridding N. Williams R. C. Woodhall N. . (2024). Implications of long-term sample storage on the recovery of viruses from wastewater and biobanking. Water Res. 265:122209. doi: 10.1016/j.watres.2024.122209, 39126986

[ref21] Feng P. Li R. F. Wang K. X. Deng H. H. Jin G. H. Chen R. F. . (2025). Shiwei Longdanhua capsule protects from hygrothermal condition and influenza infection by suppressing metabolism and inflammatory pathway. Phytomedicine 148:157343. doi: 10.1016/j.phymed.2025.157343, 41075514

[ref22] Folegatti P. M. Pepin S. Tabar C. Fries K. Talanova O. See S. . (2025). Comparative assessment of immunogenicity and safety of recombinant influenza vaccine in children, adolescents, and adults: results from a phase 3, immunobridging, open-label, non-randomised study. Lancet Infect. Dis. 25, 1097–1105. doi: 10.1016/S1473-3099(25)00153-7, 40412421

[ref23] GBD 2023 Lower Respiratory Infections and Antimicrobial Resistance Collaborators (2026). Global burden of lower respiratory infections and aetiologies, 1990-2023: a systematic analysis for the Global Burden of Disease Study. Lancet Infect. Dis. 26, 343–361. doi: 10.1016/S1473-3099(25)00689-9 41412141

[ref24] Gilbertson B. Duncan M. Subbarao K. (2023). Role of the viral polymerase during adaptation of influenza a viruses to new hosts. Curr. Opin. Virol. 62:101363. doi: 10.1016/j.coviro.2023.101363, 37672875

[ref25] Gong H. H. Worley M. J. Carver K. A. Godin C. J. Deng J. C. (2025). Deficient neutrophil responses early in influenza infection promote viral replication and pulmonary inflammation. PLoS Pathog. 21:e1012449. doi: 10.1371/journal.ppat.1012449, 39823516 PMC11845034

[ref26] Goodman K. E. Shams S. M. Magder L. S. Baghdadi J. D. Morgan D. J. Harris A. D. (2025). Generative artificial intelligence-based surveillance for avian influenza across a statewide healthcare system. Clin. Infect. Dis. 81, 900–903. doi: 10.1093/cid/ciaf369, 40801548

[ref27] Groen K. Hale B. G. (2026). Human autoantibodies against type I interferons in severe viral disease. Curr. Opin. Virol. 74:101511. doi: 10.1016/j.coviro.2026.101511, 41605119

[ref28] Grzesiak E. Bent B. Mcclain M. T. Woods C. W. Tsalik E. L. Nicholson B. P. . (2021). Assessment of the feasibility of using noninvasive wearable biometric monitoring sensors to detect influenza and the common cold before symptom onset. JAMA Netw. Open 4:e2128534. doi: 10.1001/jamanetworkopen.2021.28534, 34586364 PMC8482058

[ref29] Hayden F. G. Lenk R. P. Stonis L. Oldham-Creamer C. Kang L. L. Epstein C. (2022). Favipiravir treatment of uncomplicated influenza in adults: results of two phase 3, randomized, double-blind, placebo-controlled trials. J. Infect. Dis. 226, 1790–1799. doi: 10.1093/infdis/jiac135, 35639525 PMC9650493

[ref30] He F. Xu J. Yu T. Zhu X. Wang X. Yang J. . (2026). Traditional Chinese medicine in influenza treatment: a bibliometric analysis integrating multiple databases. Front. Microbiol. 17:1761339. doi: 10.3389/fmicb.2026.1761339, 41909248 PMC13018163

[ref31] Helanterä I. Gissler M. Rimhanen-Finne R. Ikonen N. Kanerva M. Lempinen M. . (2021). Epidemiology of laboratory-confirmed influenza among kidney transplant recipients compared to the general population-a nationwide cohort study. Am. J. Transplant. 21, 1848–1856. doi: 10.1111/ajt.16421, 33252189

[ref32] Hirotsu N. Sakaguchi H. Fukao K. Kojima S. Piedra P. A. Tsuchiya K. . (2023). Baloxavir safety and clinical and virologic outcomes in influenza virus-infected pediatric patients by age group: age-based pooled analysis of two pediatric studies conducted in Japan. BMC Pediatr. 23:35. doi: 10.1186/s12887-023-03841-5, 36681802 PMC9860230

[ref33] Hu W. Liu Y. Dong J. Peng X. Yang C. Wang H. . (2025). Evaluation of a machine learning model based on laboratory parameters for the prediction of influenza a and B in Chongqing, China: multicenter model development and validation study. J. Med. Internet Res. 27:e67847. doi: 10.2196/67847, 40373305 PMC12123241

[ref34] Hu Z. Liu H. Wu W. Ali T. Junka A. Sharopov F. S. . (2026). Artemisia extracts suppress H1N1 influenza a virus infection by targeting viral Ha/Na proteins and modulating the Tlr4/MyD88/Nf-κB signaling Axis. Pharmaceuticals (Basel) 19:275. doi: 10.3390/ph19020275, 41754817 PMC12943541

[ref35] Huang X. Zhang Y. Chen J. Yang Z. Chen M. Xue Z. . (2026). A multiplexed three-channel detection system for rapid home-based diagnosis of respiratory viruses. Talanta 297:128716. doi: 10.1016/j.talanta.2025.128716, 40848563

[ref36] Hussain S. Meijer A. Govorkova E. A. Dapat C. Gubareva L. V. Barr I. G. . (2025). Global update on the susceptibilities of influenza viruses to neuraminidase inhibitors and the cap-dependent endonuclease inhibitor baloxavir, 2020-2023. Antivir. Res. 241:106217. doi: 10.1016/j.antiviral.2025.106217, 40571063 PMC12391581

[ref37] Ishaqui A. Hayat Khan A. Sulaiman S. A. S. Taher Alsultan M. Khan I. (2021). Comparative efficacy assessment of antiviral alone and antiviral-antibiotic combination in prevention of influenza-B infection associated complications. Expert Rev. Anti-Infect. Ther. 19, 1165–1173. doi: 10.1080/14787210.2021.1889369, 33567928

[ref38] Ji Y. Liu H. Liu L. Jiang L. Bai C. Sun F. . (2026). Ma Xing Shi Gan decoction reduces influenza a virus nucleoprotein levels in association with modulation of lactate-Hmgb1 lactylation and autophagosome accumulation. J. Ethnopharmacol. 364:121540. doi: 10.1016/j.jep.2026.121540, 41850635

[ref39] Johansen N. D. Modin D. Loiacono M. M. Harris R. C. Dufournet M. Larsen C. S. . (2025). High-dose influenza vaccine effectiveness against hospitalization in older adults. N. Engl. J. Med. 393, 2291–2302. doi: 10.1056/NEJMoa2509907, 40888720

[ref40] Kainth M. K. Fishbein J. S. Aydillo T. Escalera A. Odusanya R. Grammatikopoulos K. . (2022). Obesity and metabolic dysregulation in children provide protective influenza vaccine responses. Viruses 14:124. doi: 10.3390/v14010124, 35062328 PMC8777815

[ref41] Kambayashi R. Goto A. Izumi-Nakaseko H. Takei Y. Sugiyama A. (2024). Characterization of cardiovascular profile of anti-influenza drug peramivir: a reverse-translational study using the isoflurane-anesthetized dog. J. Pharmacol. Sci. 154, 218–224. doi: 10.1016/j.jphs.2024.02.002, 38395523

[ref42] Kang Y. M. Heo G. B. An S. H. Lee H. Park E. Cha R. M. . (2024). Highly pathogenic avian influenza a(H5N1) virus infection in cats, South Korea, 2023. Emerg. Infect. Dis. 30, 2510–2520. doi: 10.3201/eid3012.240154, 39499954 PMC11616667

[ref43] Kang H. Lee J. Lee S. H. Jeon J. Mun C. Yang J. Y. . (2025). Interpretability-driven deep learning for Sers-based classification of respiratory viruses. Biosens. Bioelectron. 289:117891. doi: 10.1016/j.bios.2025.117891, 40840132

[ref44] Kildegaard H. Lund L. C. Pottegård A. Stensballe L. G. (2023). Effectiveness of the quadrivalent live attenuated influenza vaccine against influenza-related hospitalisations and morbidity among children aged 2 to 6 years in Denmark: a nationwide cohort study emulating a target trial. Lancet Child Adolesc Health 7, 852–862. doi: 10.1016/S2352-4642(23)00225-0, 37898144

[ref45] King J. P. Nguyen H. Q. Kiniry E. L. Phillips C. H. Gaglani M. Martin E. T. . (2024). Elevated body mass index is not significantly associated with reduced influenza vaccine effectiveness. Sci. Rep. 14:21466. doi: 10.1038/s41598-024-72081-z, 39271784 PMC11399117

[ref46] Kshirsagar A. Derosa D. Politza A. J. Liu T. Dong M. Guan W. (2025). Point-of-need one-pot multiplexed Rt-Lamp test for detecting three common respiratory viruses in saliva. Biosens. Bioelectron. 288:117836. doi: 10.1016/j.bios.2025.117836, 40768948 PMC12395363

[ref47] Kumar D. Ison M. G. Mira J. P. Welte T. Hwan Ha J. Hui D. S. . (2022). Combining baloxavir marboxil with standard-of-care neuraminidase inhibitor in patients hospitalised with severe influenza (flagstone): a randomised, parallel-group, double-blind, placebo-controlled, superiority trial. Lancet Infect. Dis. 22, 718–730. doi: 10.1016/S1473-3099(21)00469-2, 35085510

[ref48] Kumar N. Towers D. Myers S. Galvin C. Kireev D. Ellington A. D. . (2023). Graphene field effect biosensor for concurrent and specific detection of Sars-CoV-2 and influenza. ACS Nano 17, 18629–18640. doi: 10.1021/acsnano.3c07707, 37703454

[ref49] Lee C. W. Tai Y. L. Huang L. M. Chi H. Huang F. Y. Chiu N. C. . (2021). Efficacy of clarithromycin-naproxen-oseltamivir combination therapy versus oseltamivir alone in hospitalized pediatric influenza patients. J. Microbiol. Immunol. Infect. 54, 876–884. doi: 10.1016/j.jmii.2020.08.017, 32978076

[ref50] Lehto K. M. Länsivaara A. Hyder R. Luomala O. Lipponen A. Hokajärvi A. M. . (2024). Wastewater-based surveillance is an efficient monitoring tool for tracking influenza a in the community. Water Res. 257:121650. doi: 10.1016/j.watres.2024.121650, 38692254

[ref51] Lei B. Liu Z. Xie P. Li X. Cui Z. Chen R. . (2026). Liu Shen Wan regulates the Sphk1/S1P axis to ameliorate influenza-induced inflammation via integrated network pharmacology and lipidomics. Front. Immunol. 16:1764754. doi: 10.3389/fimmu.2025.1764754, 41624880 PMC12855086

[ref52] Leibovici Weissman Y. Cooper L. Sternbach N. Ashkenazi-Hoffnung L. Yahav D. (2021). Clinical efficacy and safety of high dose trivalent influenza vaccine in adults and immunosuppressed populations - a systematic review and meta-analysis. J. Infect. 83, 444–451. doi: 10.1016/j.jinf.2021.08.028, 34425161

[ref53] Levi Y. Gande V. Shmueli E. Patalon T. Gazit S. Brandeau M. L. . (2026). Smartwatch-derived versus self-reported outcomes of physiological recovery after Covid-19, influenza, and group a streptococcus: a 2-year prospective cohort study. Lancet Digit. Health 8:100956. doi: 10.1016/j.landig.2025.100956, 41654404 PMC12992200

[ref54] Li R. Liu Y. Li G. Zhang J. Luo Y. Zhou B. . (2025). Chebulinic acid from Chebulae fructus alleviates influenza virus-induced acute lung injury by inhibiting Ido1-Kyn axis activation. Phytomedicine 148:157362. doi: 10.1016/j.phymed.2025.157362, 41106098

[ref55] Li Y. Wang Z. Wang J. Jiang Z. Chen M. Ai H. . (2025). Arrdc4-mediated glycolysis enhances innate immunity to influenza a virus through fructose-1,6-bisphosphate. Proc. Natl. Acad. Sci. USA 122:e2512385122. doi: 10.1073/pnas.2512385122, 40875808 PMC12415272

[ref56] Liao F. Li S. Wu L. Chen J. Luo Z. Zhong M. . (2025). Herbacetin alleviates influenza virus-induced lung injury and fibrosis by targeting the neuraminidase protein. Pharmaceuticals (Basel) 18:1306. doi: 10.3390/ph18091306, 41011177 PMC12473129

[ref57] Lim J. J. Dar S. Venter D. Horcajada J. P. Kulkarni P. Nguyen A. . (2022). A phase 2 randomized, double-blind, placebo-controlled trial of the monoclonal antibody Mhaa4549A in patients with acute uncomplicated influenza a infection. Open Forum Infect. Dis. 9:ofab630. doi: 10.1093/ofid/ofab630, 35106315 PMC8801227

[ref58] Lim J. Koprowski K. Stavins R. Xuan N. Hoang T. H. Baek J. . (2024). Point-of-care multiplex detection of respiratory viruses. ACS Sens. 9, 4058–4068. doi: 10.1021/acssensors.4c00992, 39101394

[ref59] Lippi G. Sanchis-Gomar F. (2021). Cardiac troponin elevation in patients with influenza virus infections. Biom. J. 44, 183–189. doi: 10.1016/j.bj.2020.06.001, 33097442 PMC8178554

[ref60] Lu S. Shengle Q. Shiqing L. Huihuang D. Qiaolian C. Ruifeng C. . (2026). Sheng Jiangsan alleviated influenza-induced acute lung injury by regulating *Lactobacillus murinus*. J. Ethnopharmacol. 367:121524. doi: 10.1016/j.jep.2026.121524, 41932663

[ref61] Luo L. Zhang Y. Sun Z. He Y. Ma J. Yang X. . (2026). G4-Nasba: An ultra-fast, highly sensitive and portable isothermal detection system for diagnosing influenza a virus. Talanta 297:128657. doi: 10.1016/j.talanta.2025.128657, 40782741

[ref62] Ma X. Chen Q. Cai Y. Chen C. Lu J. Yang Z. . (2025). Intranasal mosaic H1N1 live attenuated influenza vaccine elicits broad cross-reactive immunity and protection against group 1 and 2 influenza a viruses. MedComm (2020) 6:e70557. doi: 10.1002/mco2.70557, 41409094 PMC12706173

[ref63] Ma X. Zhang Y. Sun L. Zhao H. Wang Z. Hao C. . (2026). Inhibition of influenza a virus infection by natural isoquinoline alkaloid neferine targeting virus nucleoprotein. Phytomedicine 152:157873. doi: 10.1016/j.phymed.2026.157873, 41619557

[ref64] Mikušová M. Tomčíková K. Briestenská K. Kostolanský F. Varečková E. (2022). The contribution of viral proteins to the synergy of influenza and bacterial co-infection. Viruses 14:1064. doi: 10.3390/v14051064 PMC9143653 35632805

[ref65] Moirangthem R. Cordela S. Khateeb D. Shor B. Kosik I. Schneidman-Duhovny D. . (2024). Dual neutralization of influenza virus hemagglutinin and neuraminidase by a bispecific antibody leads to improved antiviral activity. Mol. Ther. 32, 3712–3728. doi: 10.1016/j.ymthe.2024.07.023, 39086132 PMC11489563

[ref66] Mombelli M. Neofytos D. Huynh-Do U. Sánchez-Céspedes J. Stampf S. Golshayan D. . (2024). Immunogenicity of high-dose versus Mf59-Adjuvanted versus standard influenza vaccine in solid organ transplant recipients: the Swiss/Spanish trial in solid organ transplantation on prevention of influenza (Stop-flu trial). Clin. Infect. Dis. 78, 48–56. doi: 10.1093/cid/ciad477, 37584344 PMC10810716

[ref67] Monto A. S. Kuhlbusch K. Bernasconi C. Cao B. Cohen H. A. Graham E. . (2025). Efficacy of Baloxavir treatment in preventing transmission of influenza. N. Engl. J. Med. 392, 1582–1593. doi: 10.1056/NEJMoa2413156, 40267424

[ref68] Muramatsu H. Lam K. Bajusz C. Laczkó D. Karikó K. Schreiner P. . (2022). Lyophilization provides long-term stability for a lipid nanoparticle-formulated, nucleoside-modified mrna vaccine. Mol. Ther. 30, 1941–1951. doi: 10.1016/j.ymthe.2022.02.001, 35131437 PMC8815268

[ref69] Nazareth J. Veli N. Pan D. Martin C. A. Kekere-Ekun A. Hassan F. A. . (2025). Duration of the serological response and effectiveness of the inactivated influenza vaccine in healthy adults aged 18-65 years: a systematic review and meta-analysis. Lancet Microbe 6:101136. doi: 10.1016/j.lanmic.2025.101136, 41067240

[ref70] Nguyen-Tran H. Park S. W. Vogt M. R. Permaul P. Spaulding A. B. Hernandez M. L. . (2026). Dynamics of endemic virus re-emergence in children in the Usa following the Covid-19 pandemic (2022-23): a prospective, multicentre, longitudinal, immunoepidemiological surveillance study. Lancet Infect. Dis. 26, 22–33. doi: 10.1016/S1473-3099(25)00349-4, 40782819 PMC12458095

[ref71] Nicoli F. Cabral-Piccin M. P. Papagno L. Gallerani E. Fusaro M. Folcher V. . (2022). Altered basal lipid metabolism underlies the functional impairment of naive Cd8(+) T cells in elderly humans. J. Immunol. 208, 562–570. doi: 10.4049/jimmunol.2100194, 35031578 PMC7615155

[ref72] Okiyama S. Fukuda M. Sode M. Takahashi W. Ikeda M. Kato H. . (2022). Examining the use of an artificial intelligence model to diagnose influenza: development and validation study. J. Med. Internet Res. 24:e38751. doi: 10.2196/38751, 36374004 PMC9823578

[ref73] Owusu D. Rolfes M. A. Arriola C. S. Daily Kirley P. Alden N. B. Meek J. . (2022). Rates of severe influenza-associated outcomes among older adults living with diabetes-influenza hospitalization surveillance network (FluSurv-net), 2012-2017. Open Forum Infect. Dis. 9:ofac131. doi: 10.1093/ofid/ofac131 PMC9017364 35450083

[ref74] Park J. H. Kim B. Antigua K. J. C. Jeong J. H. Kim C. I. Choi W. S. . (2021). Baloxavir-oseltamivir combination therapy inhibits the emergence of resistant substitutions in influenza a virus pa gene in a mouse model. Antivir. Res. 193:105126. doi: 10.1016/j.antiviral.2021.105126, 34217753

[ref75] Park S. H. Kim S. C. Lee A. Park J. K. Kang E. B. Ha S. B. . (2025). Highly sensitive reusable electrochemical sensor for H1N1 influenza a virus nucleoproteins. Biosens. Bioelectron. 288:117829. doi: 10.1016/j.bios.2025.117829, 40750471

[ref76] Park B. J. Park M. S. Lee J. M. Song Y. J. (2021). Specific detection of influenza a and B viruses by Crispr-Cas12a-based assay. Biosensors 11:88. doi: 10.3390/bios11030088, 33808752 PMC8003587

[ref77] Patel M. C. Nguyen H. T. Mishin V. P. Pascua P. N. Q. Champion C. Lopez-Esteva M. . (2025). Antiviral susceptibility monitoring: testing algorithm, methods, and findings for influenza season, 2023-2024. Antivir. Res. 244:106299. doi: 10.1016/j.antiviral.2025.106299, 41083110 PMC12621569

[ref78] Peacock T. P. Sheppard C. M. Lister M. G. Staller E. Frise R. Swann O. C. . (2023). Mammalian Anp32A and Anp32B proteins drive differential polymerase adaptations in avian influenza virus. J. Virol. 97:e0021323. doi: 10.1128/jvi.00213-23, 37074204 PMC10231198

[ref79] Praphasiri P. Prasert K. Shrestha M. Ditsungnoen D. Chittaganpich M. Chawalchitiporn S. . (2023). Does prior vaccination affect the immune response to seasonal influenza vaccination among older adults? Findings from a prospective cohort study in a Northeastern Province of Thailand. PLoS One 18:e0279962. doi: 10.1371/journal.pone.0279962, 36735691 PMC9897550

[ref80] Putri W. Muscatello D. J. Stockwell M. S. Newall A. T. (2018). Economic burden of seasonal influenza in the United States. Vaccine 36, 3960–3966. doi: 10.1016/j.vaccine.2018.05.057, 29801998

[ref81] Rashidi O. Moattari A. Pirbonyeh N. Emami A. Kadivar M. R. Tavakoli Movaghar N. . (2021). Investigation of genetic variation: neuraminidase gene of influenza a virus H1N1/pdm09, shiraz, Iran (2015-2016). J. Med. Virol. 93, 4763–4772. doi: 10.1002/jmv.26894, 33605468

[ref82] Rojas R. E. Equils O. Villacian J. Mann A. Van Duijnhoven W. Vingerhoets J. . (2025). Prophylactic efficacy of Cd388, a novel drug-fc conjugate, in a human influenza a/H3N2 virus challenge model: a randomized, controlled phase 2a study. Clin. Infect. Dis. 81, e310–e318. doi: 10.1093/cid/ciaf465, 41060047 PMC12728294

[ref83] Rolfes M. A. Kniss K. Kirby M. K. Garg S. Reinhart K. Davis C. T. . (2025). Human infections with highly pathogenic avian influenza a(H5N1) viruses in the United States from march 2024 to may 2025. Nat. Med. 31, 3889–3898. doi: 10.1038/s41591-025-03905-2, 40712649 PMC12477757

[ref84] Rose A. M. C. Nicolay N. Mazagatos C. Martínez-Baz I. Launay O. De Mot L. . (2025). Vaccine effectiveness against influenza a in older adults and the effect of chronic conditions: results from the I-move and Vebis multicentre European hospital case-control studies, 2015/16-2023/24. BMC Med. 23:602. doi: 10.1186/s12916-025-04426-y PMC12581461 41185010

[ref85] Saito R. Chon I. Sato I. Kawashima T. Kakuya F. Saito T. . (2025). Impact of polymerase acidic subunit (pa) substitutions on fever and symptoms in pediatric influenza a patients treated with baloxavir: a pooled four-season analysis. J. Infect. 91:106555. doi: 10.1016/j.jinf.2025.106555, 40684885

[ref86] Salmanton-García J. Marchesi F. Navrátil M. Piukovics K. Del Principe M. I. Criscuolo M. . (2025). Respiratory viruses in patients with hematological malignancy in boreal autumn/winter 2023-2024: Epicovideha-Epiflueha report. Am. J. Hematol. 100, 358–374. doi: 10.1002/ajh.27565, 39715069 PMC11803548

[ref87] Sanchez Ruiz M. A. Marques D. F. Lomholt F. K. Vestergaard L. S. Monge S. Lozano Álvarez M. . (2025). Surveillance of severe acute respiratory infections associated with Sars-CoV-2, influenza virus and Rsv using Icd-10 codes: a case definition accuracy study across five European countries, 2021 to 2023. Euro Surveill. 30:2400748. doi: 10.2807/1560-7917.ES.2025.30.27.2400748, 40642769 PMC12262114

[ref88] Shang X. Jia J. Yu J. Zou S. Wang Z. Xu J. . (2025). Monocyte-derived macrophages support alveolar regeneration via oncostatin M post-H1N1 infection during the recovery phase. Respir. Res. 26:285. doi: 10.1186/s12931-025-03359-7, 41063200 PMC12509380

[ref89] Shen X. Yin F. (2021). The mechanisms and clinical application of traditional Chinese medicine Lianhua-Qingwen capsule. Biomed. Pharmacother. 142:111998. doi: 10.1016/j.biopha.2021.111998, 34385103 PMC8352581

[ref90] Shi H. Zhang X. Ge P. Meliopoulos V. Freiden P. Livingston B. . (2024). Inactivated influenza virus vaccines expressing Cobra hemagglutinin elicited broadly reactive, long-lived protective antibodies. Hum. Vaccin. Immunother. 20:2356269. doi: 10.1080/21645515.2024.2356269, 38826029 PMC11152115

[ref91] Shinde V. Cho I. Plested J. S. Agrawal S. Fiske J. Cai R. . (2022). Comparison of the safety and immunogenicity of a novel matrix-M-adjuvanted nanoparticle influenza vaccine with a quadrivalent seasonal influenza vaccine in older adults: a phase 3 randomised controlled trial. Lancet Infect. Dis. 22, 73–84. doi: 10.1016/S1473-3099(21)00192-4, 34563277

[ref92] Shinjoh M. Sugaya N. Yamaguchi Y. Ookawara I. Nakata Y. Narabayashi A. . (2021). Influenza vaccine effectiveness against influenza a in children based on the results of various rapid influenza tests in the 2018/19 season. PLoS One 16:e0249005. doi: 10.1371/journal.pone.0249005, 33770132 PMC7997015

[ref93] Silverman A. Walsh R. Santoro J. D. Thomas K. Ballinger E. Fisher K. S. . (2025). Influenza-associated acute necrotizing encephalopathy in us children. JAMA 334, 692–701. doi: 10.1001/jama.2025.11534, 40736730 PMC12311823

[ref94] Skaarup K. G. Davidovski F. S. Durukan E. Modin D. Lassen M. C. H. Dons M. . (2025a). Cardiac characteristics of hospitalized influenza patients: an interim analysis from the FluHeart study. Influenza Other Respir. Viruses 19:e70067. doi: 10.1111/irv.70067 PMC11835437 39965617

[ref95] Skaarup K. G. Johansen N. D. Modin D. Loiacono M. M. Harris R. C. Dufournet M. . (2025b). High-dose versus standard-dose influenza vaccine in heart failure: a prespecified analysis of the Danflu-2 trial. Circ. Heart Fail. 18:e013678. doi: 10.1161/circheartfailure.125.013678, 40884411

[ref96] Smith C. L. Bednarchik B. Aung H. Wilk D. J. Boxer R. S. Daddato A. E. . (2023). Humoral and cellular immunity induced by Adjuvanted and standard trivalent influenza vaccine in older nursing home residents. J. Infect. Dis. 228, 704–714. doi: 10.1093/infdis/jiad071, 36951196

[ref97] Sojati J. Parks O. B. Eddens T. Lan J. Johnson M. Williams J. V. (2025). Limited nasal IFN production contributes to delayed respiratory virus clearance and suboptimal vaccine responses. JCI Insight 10:e18283. doi: 10.1172/jci.insight.182836, 40956633 PMC12581673

[ref98] Staller E. Carrique L. Swann O. C. Fan H. Keown J. R. Sheppard C. M. . (2024). Structures of H5N1 influenza polymerase with Anp32B reveal mechanisms of genome replication and host adaptation. Nat. Commun. 15:4123. doi: 10.1038/s41467-024-48470-3, 38750014 PMC11096171

[ref99] Su C. P. Chan K. A. Huang C. T. Fang C. T. (2022). Inhaled Zanamivir vs Oral oseltamivir to prevent influenza-related hospitalization or death: a Nationwide population-based quasi-experimental study. Clin. Infect. Dis. 75, 1273–1279. doi: 10.1093/cid/ciac217, 35299245

[ref100] Sun J. Kuai L. Zhang L. Xie Y. Zhang Y. Li Y. . (2024). Ns2 induces an influenza a Rna polymerase hexamer and acts as a transcription to replication switch. EMBO Rep. 25, 4708–4727. doi: 10.1038/s44319-024-00208-4, 39026012 PMC11549089

[ref101] Takashita E. Wolfe C. R. Jones J. C. (2025). Laboratory methods for monitoring influenza antiviral resistance. J. Infect. Dis. 232, S273–S285. doi: 10.1093/infdis/jiaf299, 41102606

[ref102] Thompson A. J. Paulson J. C. (2021). Adaptation of influenza viruses to human airway receptors. J. Biol. Chem. 296:100017. doi: 10.1074/jbc.REV120.013309, 33144323 PMC7948470

[ref103] Tian M. Ma J. Li Z. Ye C. Cui X. Wang G. . (2025). Senescent cells promote viral infection-associated inflammation and tissue damage through a robust Nf-κB pathway. Cell Commun. Signal 23:464. doi: 10.1186/s12964-025-02466-8, 41152908 PMC12560573

[ref104] Tsunetsugu-Yokota Y. Ito S. Adachi Y. Onodera T. Kageyama T. Takahashi Y. (2022). Saliva as a useful tool for evaluating upper mucosal antibody response to influenza. PLoS One 17:e0263419. doi: 10.1371/journal.pone.0263419, 35130308 PMC8820602

[ref105] Varughese J. E. Jacob A. A. Thampi A. John J. J. David A. John G. M. . (2025). Utility of saliva for detecting influenza a virus by cost-effective extraction-free Sybr green-based Pcr. J. Med. Virol. 97:e70475. doi: 10.1002/jmv.70475, 40590709

[ref106] Wang Y. Wang H. Zhang Y. Ma A. Liu D. Li X. . (2025). Single-dose suraxavir marboxil for acute uncomplicated influenza in adults and adolescents: a multicenter, randomized, double-blind, placebo-controlled phase 3 trial. Nat. Med. 31, 639–646. doi: 10.1038/s41591-024-03419-3, 39775042

[ref107] Wen T. Arditi B. Riley L. E. Sobhani N. C. Norton M. D'alton M. . (2021). Influenza complicating delivery hospitalization and its association with severe maternal morbidity in the United States, 2000-2018. Obstet. Gynecol. 138, 218–227. doi: 10.1097/AOG.0000000000004462, 34237767

[ref108] Widge A. T. Hofstetter A. R. Houser K. V. Awan S. F. Chen G. L. Burgos Florez M. C. . (2023). An influenza hemagglutinin stem nanoparticle vaccine induces cross-group 1 neutralizing antibodies in healthy adults. Sci. Transl. Med. 15:eade4790. doi: 10.1126/scitranslmed.ade4790, 37075129 PMC10619166

[ref109] Wu X. Su Y. Xu Y. Hu Q. Jiao J. Ma J. . (2025). A bioinspired microfluidic nucleic acid sensing platform for rapid and simultaneous screening of viral respiratory infections. ACS Sens. 10, 4634–4645. doi: 10.1021/acssensors.5c01021, 40440480

[ref110] Xiao Y. Chen J. Hou X. Wang H. Zhang K. Xu S. . (2025). A NanoLock-enabled, Craspase-based strategy for highly sensitive Rna detection. Nucleic Acids Res. 53:gkaf907. doi: 10.1093/nar/gkaf907, 40966498 PMC12445650

[ref111] Xiong S. Guo Y. Jiang L. Wang Y. Chen W. Bai Z. . (2025). A simple, rapid, and cost-effective model for predicting critical influenza a infection in children: a multicentre, retrospective cohort study. BMC Pediatr. 25:746. doi: 10.1186/s12887-025-06085-7, 41039296 PMC12492913

[ref112] Xu A. Xie L. Zhong L. Zhao X. Wan Z. Wang J. H. . (2026). Development of a multiplex real-time RT-PCR assay for simultaneous detection of 18 respiratory viruses. Front. Cell. Infect. Microbiol. 15:1727780. doi: 10.3389/fcimb.2025.1727780, 41602116 PMC12833288

[ref113] Xu M. Cai T. Yue T. Zhang P. Huang J. Liu Q. . (2023). Comparative effectiveness of oseltamivir versus peramivir for hospitalized children (aged 0-5 years) with influenza infection. Int. J. Infect. Dis. 128, 157–165. doi: 10.1016/j.ijid.2022.12.043, 36608788

[ref114] Yan Y. Xu J. Chen Z. Xu Y. Qin L. Zhao L. . (2025). Heat shock protein A1L restricts influenza a virus by ubiquitination of Na. J. Virol. 99:e0077125. doi: 10.1128/jvi.00771-25, 40788012 PMC12456019

[ref115] Yang L. Li C. Li Y. Sun X. Mao H. Zou Z. . (2026). Methanol extract of Artemisia argyi against influenza a virus via inhibition of Pi3K-Akt pathway activation. J. Ethnopharmacol. 358:120973. doi: 10.1016/j.jep.2025.120973, 41317814

[ref116] Yang Z. Zhan Y. Li Z. Lin Z. Fang Z. Li H. . (2025). Efficacy and safety of onradivir in adults with acute uncomplicated influenza a infection in China: a multicentre, double-blind, randomised, placebo-controlled and oseltamivir-controlled, phase 3 trial. Lancet Respir. Med. 13, 597–610. doi: 10.1016/S2213-2600(25)00046-3, 40489986

[ref117] Yoshida A. Uekusa Y. Suzuki T. Bauer M. Sakai N. Yamauchi Y. (2025). Enhanced visualization of influenza a virus entry into living cells using virus-view atomic force microscopy. Proc. Natl. Acad. Sci. USA 122:e2500660122. doi: 10.1073/pnas.2500660122, 40966294 PMC12478160

[ref118] Zafeiriadou A. Georgakopoulou G. Pitaouli F. Thomaidis N. Markou A. (2025). One assay, nine targets: advancing viral surveillance with multiplex Rt-ddpcr. Anal. Chem. 97, 22229–22237. doi: 10.1021/acs.analchem.5c04372, 41042156 PMC12529474

[ref119] Zhang D. Li A. Li C. Ren P. Zhang L. Liu B. . (2025). A fully automated rotary microfluidic platform for high-throughput multiplex detection of respiratory tract pathogens. Microsyst Nanoeng 11:186. doi: 10.1038/s41378-025-01044-9, 41087332 PMC12521484

[ref120] Zhang J. Wu B. Yang N. Lin X. Chen M. Chang H. . (2025). The mechanism of action in Mussaenda pubescens (Yuye Jinhua) against influenza a virus: evidence from in vitro and in vivo studies. Phytomedicine 145:157070. doi: 10.1016/j.phymed.2025.157070, 40684492

[ref121] Zhang X. Zhang Y. Wei F. (2024). Research progress on the nonstructural protein 1 (Ns1) of influenza a virus. Virulence 15:2359470. doi: 10.1080/21505594.2024.2359470, 38918890 PMC11210920

[ref122] Zhu W. Gu L. (2024). Resurgence of seasonal influenza driven by a/H3N2 and B/Victoria in succession during the 2023-2024 season in Beijing showing increased population susceptibility. J. Med. Virol. 96:e29751. doi: 10.1002/jmv.29751, 38884384

[ref123] Zhu Y. Sun Y. Deng X. Cao P. Li S. Yu H. . (2025). Matrix protein 1 (M1) of influenza a virus: structural and functional insights. Emerg Microbes Infect 14:2558881. doi: 10.1080/22221751.2025.2558881, 40925098 PMC12451964

